# Molecular dissection of the replication system of plasmid pIGRK encoding two in-frame Rep proteins with antagonistic functions

**DOI:** 10.1186/s12866-019-1595-3

**Published:** 2019-11-13

**Authors:** Paweł Wawrzyniak, Agnieszka Sobolewska-Ruta, Piotr Zaleski, Natalia Łukasiewicz, Paulina Kabaj, Piotr Kierył, Agata Gościk, Anna Bierczyńska-Krzysik, Piotr Baran, Anna Mazurkiewicz-Pisarek, Andrzej Płucienniczak, Dariusz Bartosik

**Affiliations:** 1 Bioengineering Department, Institute of Biotechnology and Antibiotics, Starościńska 5, 02-516 Warsaw, Poland; 2 Department of Bacterial Genetics, Institute of Microbiology, Faculty of Biology, University of Warsaw, Ilji Miecznikowa 1, 02-096 Warsaw, Poland

**Keywords:** Overlapping genes, Plasmid replication, Replication system, Rep protein, In-frame proteins, Transcription regulation, *Klebsiella pneumoniae*

## Abstract

**Background:**

Gene overlapping is a frequent phenomenon in microbial genomes. Excluding so-called “trivial overlapping”, there are significant implications of such genetic arrangements, including regulation of gene expression and modification of protein activity. It is also postulated that, besides gene duplication, the appearance of overlapping genes (OGs) is one of the most important factors promoting a genome’s novelty and evolution. OGs coding for in-frame proteins with different functions are a particularly interesting case. In this study we identified and characterized two in-frame proteins encoded by OGs on plasmid pIGRK from *Klebsiella pneumoniae*, a representative of the newly distinguished pHW126 plasmid family.

**Results:**

A single *repR* locus located within the replication system of plasmid pIGRK encodes, in the same frame, two functional polypeptides: a full-length RepR protein and a RepR’ protein (with *N*-terminal truncation) translated from an internal START codon. Both proteins form homodimers, and interact with diverse DNA regions within the plasmid replication origin and *repR* promoter operator. Interestingly, RepR and RepR’ have opposing functions – RepR is crucial for initiation of pIGRK replication, while RepR’ is a negative regulator of this process. Nevertheless, both proteins act cooperatively as negative transcriptional regulators of their own expression.

**Conclusions:**

Regulation of the initiation of pIGRK replication is a complex process in which a major role is played by two in-frame proteins with antagonistic functions. In-frame encoded Rep proteins are uncommon, having been described in only a few plasmids. This is the first description of such proteins in a plasmid of the pHW126 family.

## Background

Overlapping genes (OGs) were first reported in viral genomes in the late 1970s [1]. It quickly became apparent that such gene arrangements are widespread, and also occur in bacterial and eukaryotic genomes, including the human genome [2, 3].

Genes may overlap in various configurations. They are classified in different ways, depending on whether (i) one gene is entirely located in the other gene or they only partially overlap, (ii) two genes occupy the same open reading frame (ORF) (in-frame overlapping) or different ORFs (out-of-frame overlapping), or (iii) they overlap in a convergent or divergent manner [2, 4].

Gene arrangement is an important factor affecting the regulation of gene expression [5]. Many studies have focused on divergent OGs encoding regulatory proteins and antisense RNAs [6, 7]. Far less is known about convergent OGs encoding, in a single *locus*, two in-frame proteins of different length and function [8, 9]. In a few cases it was demonstrated that a shorter protein, translated from an internal START codon, exhibits regulatory properties and is able to modulate or inhibit the activity of the full-length protein [2, 10, 11]. An interesting model for such studies are the replication initiation proteins (Reps), encoded by three broad-host-range (BHR) plasmids: RK2, R6K and RSF1010.

RK2 and R6K replicate according to the classical theta mode (characterized by replication bubbles formation, continuous replication of the leading strand and discontinuous replication of the lagging strand). In the case of RK2, a single *locus*, *trfA*, encodes two in-frame Rep proteins, namely (i) TrfA-44 (44 kDa) – the full length protein initiating plasmid replication in *Pseudomonas aeruginosa* (the *N*-terminal part of this protein contains a domain responsible for direct recruitment of DnaB helicase in this bacterium), and (ii) TrfA-33 (33 kDa) – a shorter protein, lacking the *N*-terminal region present in TrfA-44. TrfA-33 mediates replication initiation in *Escherichia coli*, where the recruitment of DnaB helicase is driven by the cell’s DnaA protein [12, 13].

Plasmid R6K also carries overlapping genes encoding π^35.5^ (35.5 kDa) and π^30.5^ (30.5 kDa) proteins (the latter lacking the *N*-terminal 37 amino acids presented in π^35.5^ [14]). π^35.5^ initiates plasmid replication, while the shorter protein, π^30.5^, is a negative regulator of this process [15].

Another interesting case is plasmid RSF1010, whose replication proceeds according to the strand-displacement mode characterized by continuous replication of both DNA strands [16]. RSF1010 encodes three Rep proteins that are indispensable for replication: RepC (initiatory protein), RepA (helicase) and a multifunctional protein RepB. RepB, also called MobA, is the full-length product of the *repB*/*mobA* gene. This protein is responsible for (i) plasmid vegetative replication (due to its primase activity) and (ii) plasmid conjugal transfer (relaxase/primase activities). A shorter protein (RepB′), translated using an alternative START codon, exhibits only primase activity and is crucial for plasmid replication [17, 18].

In this study, another example of in-frame Rep proteins was characterized. These are encoded by pIGRK (2348 bp), a narrow-host- range (NHR) plasmid originating from *Klebsiella pneumoniae* 287-w, a pathogenic strain isolated in The Children’s Memorial Health Institute in Poland [19]. pIGRK represents a newly distinguished plasmid family, whose archetype, pHW126 of *Rahnella inusitata* WMR126, is believed to replicate using the rolling circle mode (RCR) [20]. Plasmid pIGRK encodes two functional Rep proteins, RepR and RepR’, and the aim of this study was to examine their role in the initiation of plasmid replication.

## Results

### Components of the REP module of pIGRK

**Fig. 1:**
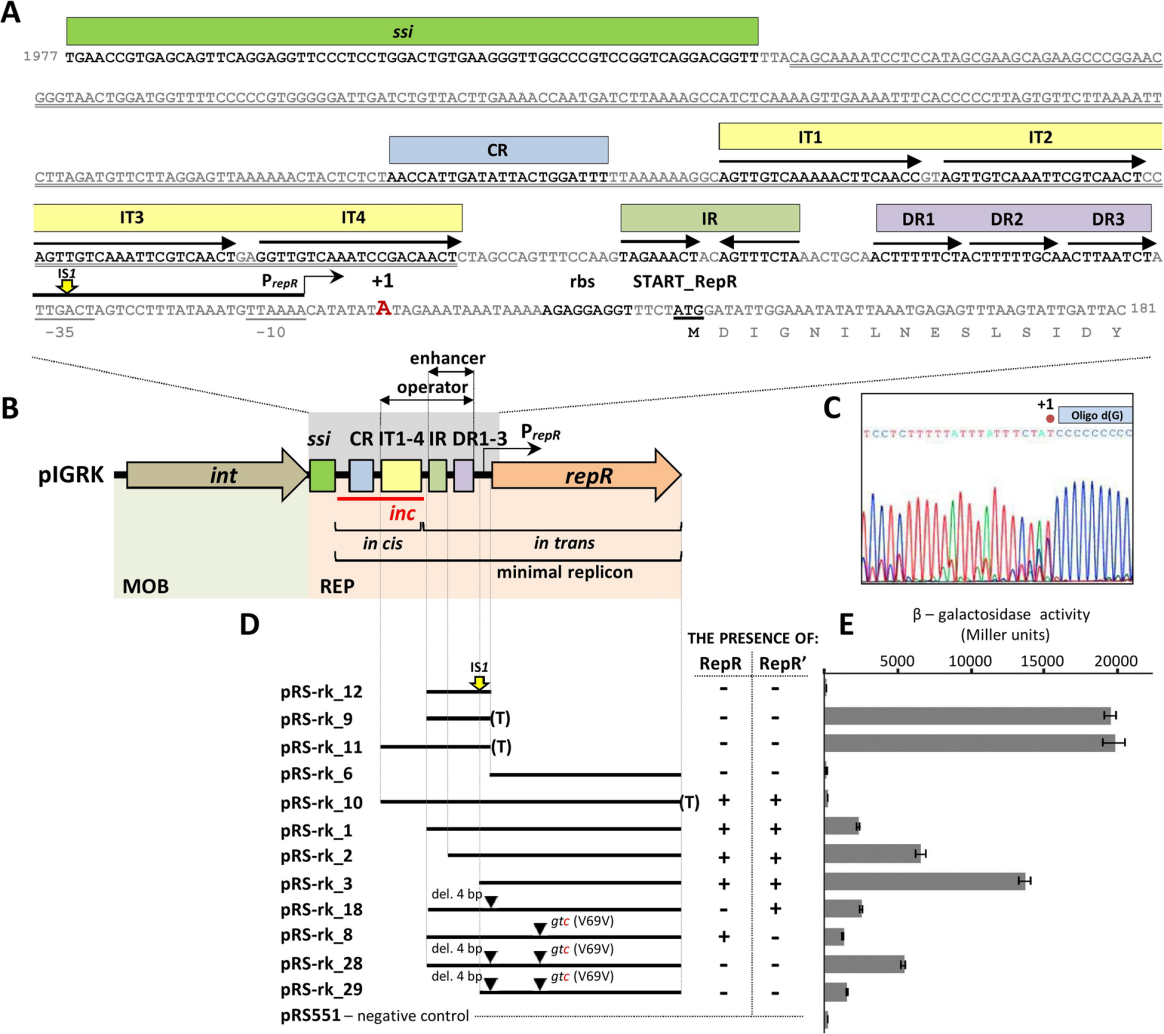
Functional analysis of the replication system of plasmid pIGRK. **a** Nucleotide sequence of the REP elements: single-strand initiation site (*ssi*), conserved region (CR), iteron-like sequences (IT), inverted repeats (IR), short direct repeats (DR) and proximal part of the *repR* gene. Predicted P_*repR*_ hexamers − 10 and − 35 (highlighted by single underlining) and the transcription start site (+ 1, in red) are indicated. The yellow arrow marks the IS*1* integration site. The putative ribosome binding site is in bold and the RepR START codon (*atg*) is in bold and underlined. The double underlined sequence indicates the DNA region indispensable *in cis* for replication. The nucleotide coordinate numbering is compatible with GenBank accession AY543071.1. **b** Genetic organization of pIGRK. Elements indispensable *in cis* and *in trans* for replication are indicated. Operator and enhancer elements of the P_*repR*_ promoter are indicated. For more detail see [Additional file 1, Figure S1]. **c** Sequencing result for the *repR* 5′-RACE product. The chromatogram represents the *repR* template strand. The oligo d(G) primer sequence is indicated and the predicted *repR* transcription start site is marked by + 1. **d** Analysis of P_*repR*_ activity and regulation. Lines represent DNA fragments of pIGRK and their mutated versions. Yellow arrow marks the IS*1* integration site. (T) – T*pro*/T*lyz* transcriptional terminator of P1, del. 4 bp – frameshift mutation introduced within the HindIII site, *gtc*(V69 V) – single nucleotide mutation (in red). pRS551 – “empty” test vector. **e** β-Galactosidase activity in strains carrying the constructs described in panel (**d)**, reflecting the strength of the promoter

Plasmid pIGRK contains two genetic modules, responsible for the initiation of replication (REP) and mobilization for conjugal transfer (MOB) (Fig. 1b) [19]. The REP module (highly similar to the REP of pHW126, both in genetic organization and sequence [21]) contains: (i) a palindromic sequence similar to single-strand initiation sites for priming DNA replication (*ssi*) found in diverse replicons [22, 23], (ii) a short (about 20 bp) DNA region, named the CR (conserved region), characteristic for all members of the pHW126-like plasmid family, (iii) four 20-bp-long direct repeats (IT1–4 – separated by 2 nucleotide spacers), similar to iterons of *theta* replication plasmids, (iv) a single inverted repeat IR (8 bp) not found in pHW126, (v) three short (9 bp) imperfect direct repeats (DR1–3) and (vi) the *repR* gene encoding a putative replication initiator protein (RepR) (Fig. 1a, b).

Previous analyses of pHW126, performed by Rozhon and co-workers [21], defined a minimal replicon of this plasmid as well as *cis*-required (*origin*) and *trans*-acting (replication initiation factor) components of its replication system. In the initial stage of this study, the REP module of pIGRK was subjected to analogous analyses.

To delineate a minimal DNA region of pIGRK capable of autonomous replication, several deletion derivatives of this plasmid were constructed and their replication abilities were tested in *E. coli* cells. This analysis revealed that all but one (*ssi*) of the aforementioned REP components are necessary for replication. The presence of *ssi* was not obligatory, although its absence caused an increase in copy number of the constructed plasmid and its rapid multimerization, which greatly reduced the stability of this replicon in bacterial cells Additional file 1: Figure S1).

The *origin* of pIGRK replication was defined using a two-plasmid system. Selected parts of the REP region were amplified by PCR and ligated to a DNA cassette containing a kanamycin resistance gene and *ori*γ of plasmid R6K (the presence of *ori*γ enabled replication of the constructed plasmids in *E. coli* DH5αλ*pir*, providing the π protein of plasmid R6K that initiates replication at *or*iγ). The constructed plasmids were then introduced into *E. coli* DH5α (lacks the *pir* gene) carrying plasmid pUC-*repR*_1, as a source of the pIGRK RepR protein. The introduced plasmids were able to replicate only when they contained a functional pIGRK *origin*, recognized by the *trans*-acting RepR protein. This analysis revealed that the *origin* comprises two components, CR and IT1–4, cloned in construct pRK-3_2γ [Additional file 1: Figure S1]. This region, when inserted into vector pUC18 (compatible with pIGRK) and introduced into an *E. coli* strain containing plasmid pRK-1 (Km^r^ derivative of pIGRK) caused removal of the residing parental replicon. Therefore, the pIGRK *origin* contains a determinant of incompatibility (*inc*), which confirmed its important regulatory functions.

Details of these analyses of the pIGRK REP module are presented in [Additional file 1: Figure S1] and summarized in Fig. 1b. We also estimated pRK-1 copy number and revealed that pIGRK is a low-copy number plasmid [see Additional file 2]. The obtained data are consistent with the results of previous analyses performed on the model pHW126 replicon [21, 24], which suggests that specific features of the REP regions might be common for all pHW126-like plasmids.

### A single *repR* locus encodes two proteins

**Fig. 2:**
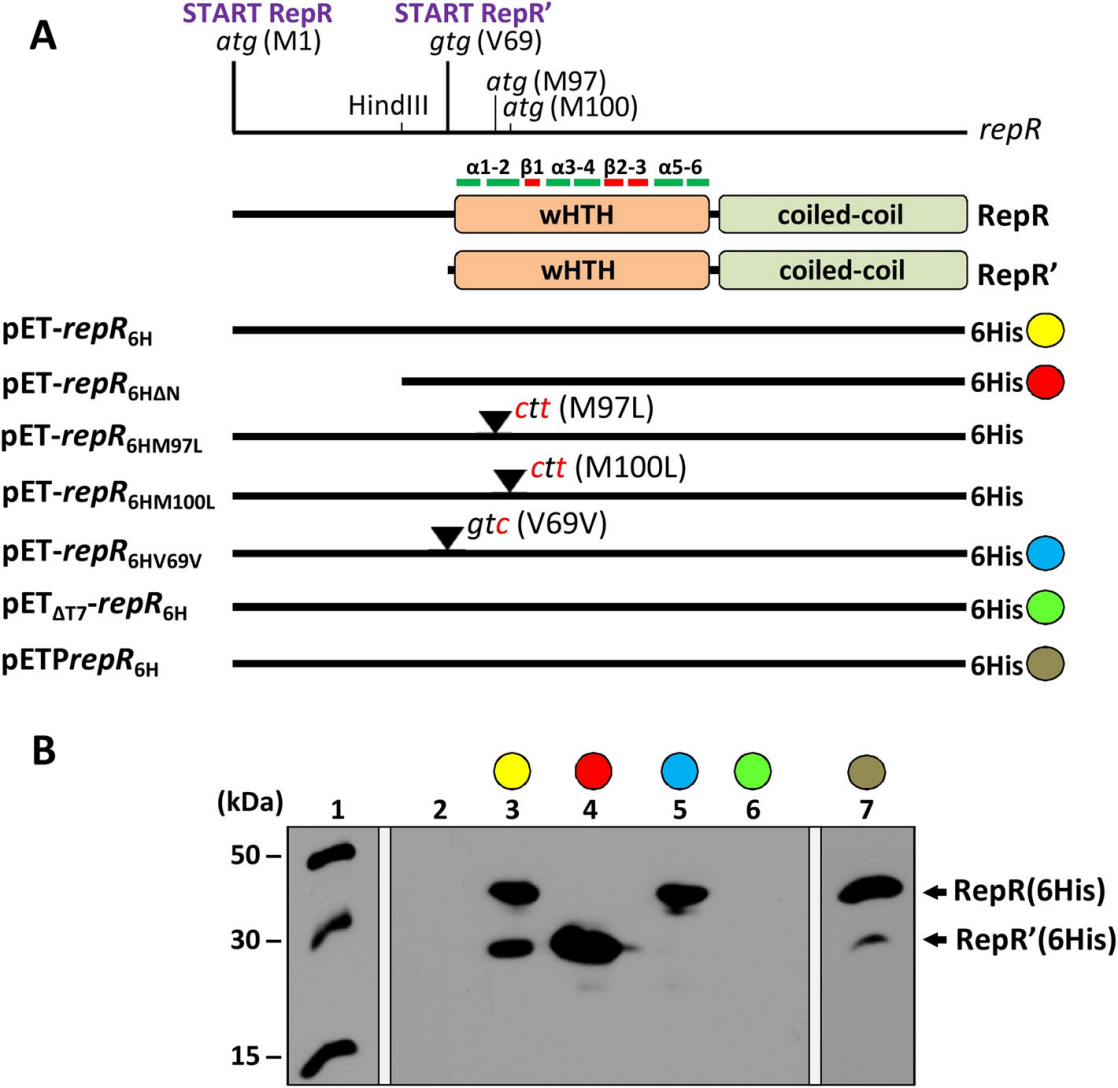
Identification of proteins encoded by the *repR* locus of pIGRK. **a** Diagram of the *repR* locus with the position of the authentic START codon and other putative internal start codons marked, plus the location of the HindIII site used to produce a frameshift mutation. Schematic depictions of the RepR and RepR’ protein sequences with putative wHTH and coiled-coil motifs shown. Within the wHTH motif, alpha helices (α) and beta sheets (β) are indicated. Lines represent *repR* variants cloned in vector pET28b + in a translational fusion with a histidine tag (6His), with some color coded. Point mutations to alter the putative internal start codons are marked in red. **b** Western blot analysis: (1) protein molecular-weight size marker, (2–7) C-terminally His-tagged proteins detected in protein cell extracts of *E. coli* strains carrying pET28b+, (2) the color-coded constructs described in the panel A (3–7). Plasmid pET_ΔT7_-*repR*_6H_ carries a wild-type copy of the *repR* gene that is not fused to the T7 promoter; pETP*repR*_6H_ carries the *repR* gene under control of its native P_*repR*_ promoter

In silico analysis of the predicted amino acid (aa) sequence of pIGRK RepR revealed that this protein does not contain sequence motifs characteristic for RCR initiator proteins of the HUH or Rep_*trans* families [25]. Using the BLAST server, a helix-turn-helix (HTH) motif was identified in the central part of the protein. The predicted secondary structure of aa sequences surrounding this putative HTH motif resembles wHTH (winged helix-turn-helix) DNA binding/dimerization motifs of MarR (multiple antibiotic resistances regulator)-like transcriptional regulators [26]. In addition, a putative coiled-coil dimerization motif located in the *C*-terminal part of RepR [27] was identified using Yaspin software (Fig. 2a).

In order to analyze interactions of RepR with DNA, the *repR* gene was amplified by PCR and cloned into expression vector pET28b+. The obtained pET-*repR*_6H_ construct was introduced into *E. coli* BL21(DE3) to overproduce recombinant RepR with a *C*-terminal histidine tag (6His) (Fig. 2a). Expression of the *repR* gene was induced by IPTG and the bacterial cell lysate was analyzed by SDS-PAGE. Surprisingly, two overproduced protein bands were detected: one corresponding in size to RepR (6His) (30.37 kDa) and a significantly smaller second, with a molecular weight of about 20 kDa.

Western blot analysis using His-Tag Antibody confirmed that both proteins contain a histidine tag and thus forms of RepR with an intact *C*-terminus (Fig. 2b). This suggested that the *repR* locus might encode two in-frame proteins – RepR and a shorter version, named RepR’ – presumably by the use of different initiator codons. Alternatively, RepR’ might be a product of proteolysis of RepR. To determine the origin of this short form, plasmid pET-*repR*_6HΔN_ was created, carrying a 5′-truncated version of *repR,* that is unable to produce full-length RepR (6His). However, this mutated *repR* gene was still able to confer production of RepR’, which clearly showed that the *repR* gene encodes two forms of the Rep protein (Fig. 2).

In the pET-*repR*_6H_ and pET-*repR*_6HΔN_ plasmids the *repR* gene was controlled by an exogenous T7 promoter. To prove that RepR’(6His) is also produced when the *repR* gene is controlled by its native promoter, pETP*repR*_6H_ plasmid was constructed. This plasmid is a pET28b + derivative deprived of the T7 promoter, with a cloned DNA fragment of pIGRK containing P_*repR*_ promoter (without operatory elements) and the *repR* gene in transcriptional fusion with 6His tag. Western blot analysis of proteins from the cell extract of *E. coli* BL21(DE3) harboring pETP*repR*_6H_ confirmed the presence of both RepR (6His) and RepR’(6His) proteins (Fig. 2b).

Three potential translation initiation sites for RepR’ were identified in the *repR* sequence. These are two methionine codons (*atg*), M97 and M100, and a codon for valine (*gtg*) - V69 (Fig. 2a). To determine which of these is responsible for the translation of RepR’, three further variants of the pET-*repR*_6H_ plasmid were prepared, carrying mutations in individual codons: (i) pET-*repR*_6HM97L_ (*atg* → *ctg*, M97 L), (ii) pET-*repR*_6HM100L_ (*atg* → *ctg*, M100 L) and (iii) pET-*repR*_6HV69V_ (*gtg* → *gtc*, V69 V) (Fig. 2a). Recombinant proteins produced by these mutated genes were detected by Western blotting. Only the V69V mutation, prevented the synthesis of RepR’(6His) (Fig. 2b). In other tested mutants, both RepR (6His) and RepR’(6His) proteins were produced. This result showed that the 69th RepR codon (*gtg*) is the internal start site for RepR’ synthesis (Fig. 2).

If one *locus* encodes two proteins, an additional internal promoter, allowing expression of the shorter gene, may be present [5, 8]. To test whether such a promoter drives the transcription of *repR’*, the plasmid pET_ΔT7_-*repR*_6H_ was constructed, lacking the phage T7 promoter that is crucial for *repR* expression. In the absence of the T7 promoter, neither RepR (6His) nor RepR’(6His) was produced, which indicated the lack of internal promoters.

### RepR and RepR’ play opposing roles in pIGRK replication initiation

In order to investigate the role of the identified in-frame RepR proteins in pIGRK replication, the aforementioned two-plasmid system (pUC-*repR*_1 and pIGRK-1_5γ) was applied. This was first used to examine whether RepR or RepR’ were able to initiate replication of pIGRK-1_5γ, containing a functional pIGRK *origin*, but lacking the *repR* gene. For this experiment, two pUC-*repR*_1 plasmid derivatives were constructed: (i) pUC-*repR*_2, providing RepR *in trans* (*gtg* RepR’ start codon replaced by *gtc* – V69V), and (ii) pUC-*repR*_3, producing RepR’ *in trans* (4-bp deletion at the HindIII restriction site of *repR*, resulting in a frame-shift mutation blocking RepR expression). The results of this analysis revealed that RepR alone was sufficient to initiate pIGRK-1_5γ replication, while RepR’ (in the absence of RepR) was unable to do so.

The copy number of pIGRK-1_5γ in the presence of either pUC-*repR*_1 or pUC-*repR*_2 was then examined using Real-Time PCR. When both RepR and RepR’ were produced *in trans* (pUC-*repR*_1), the estimated copy number of pIGRK-1_5γ was 2.3 (relative to parental pRK-1 plasmid, 1 copy), while when only RepR was supplied (pUC-*repR*_2), an increase in copy number of about two-fold was observed (5). These data suggested that the initiation of pIGRK-1_5γ replication proceeded more efficiently in the absence of RepR’, and this increased the copy number of the plasmid.

To further explore the influence of the two RepR forms on pIGRK replication, the effect of overproduction of RepR or RepR’ on the copy number of plasmid pRK-1 (pIGRK with the Km^r^ cassette) was examined. For this purpose, appropriate forms of the *repR* gene were cloned into the expression vector pBAD33 to produce the constructs pBAD-*repR*_V69V_ (RepR source) and pBAD-*repR*_ΔN_ (RepR’ source). These plasmids (and pBAD33 as a control) were introduced independently into the *E. coli* MC1061 (*ara*-) strain harboring pRK-1. The resulting strains were grown in LB medium in the presence of appropriate selective antibiotics and arabinose, which induced expression of the *repR* genes *in trans*. In this test system, the relative copy number of pRK-1 in the control strain (carrying “empty” pBAD33) was estimated at 1. In comparison, when RepR was overproduced (pBAD-*repR*_V69V_) the pRK-1 copy number increased 5-fold (5), while overproduction of RepR’ (pBAD-*repR*_ΔN_) caused a 5-fold decrease in copy number (0.2). This result confirmed that RepR and RepR’ play opposing roles at the initiation stage of pIGRK replication.

The negative influence of RepR’ on pIGRK replication initiation was observed upon overproduction of this protein. To examine the significance of RepR’ in the biology of pIGRK, functioning of the native REP module in the absence of this protein was examined. For this purpose, the *repR* gene of plasmid pRK-1 was mutated *in cis* to remove the RepR’ start codon (*gtg → gtc*, V69V). The obtained construct pRK-14 (unable to produce RepR’) and pRK-1 (control) were introduced independently into the *E. coli* strain DH5α, and the copy number of the individual plasmids was determined as before. In this case, the copy number of pRK-14 was slightly increased (1.6) compared to parental pRK-1 (1).

The observations described above clearly proved that RepR’ participates in the negative regulation of pIGRK replication initiation.

### RepR and RepR’ form dimers in vivo and in vitro

**Fig. 3:**
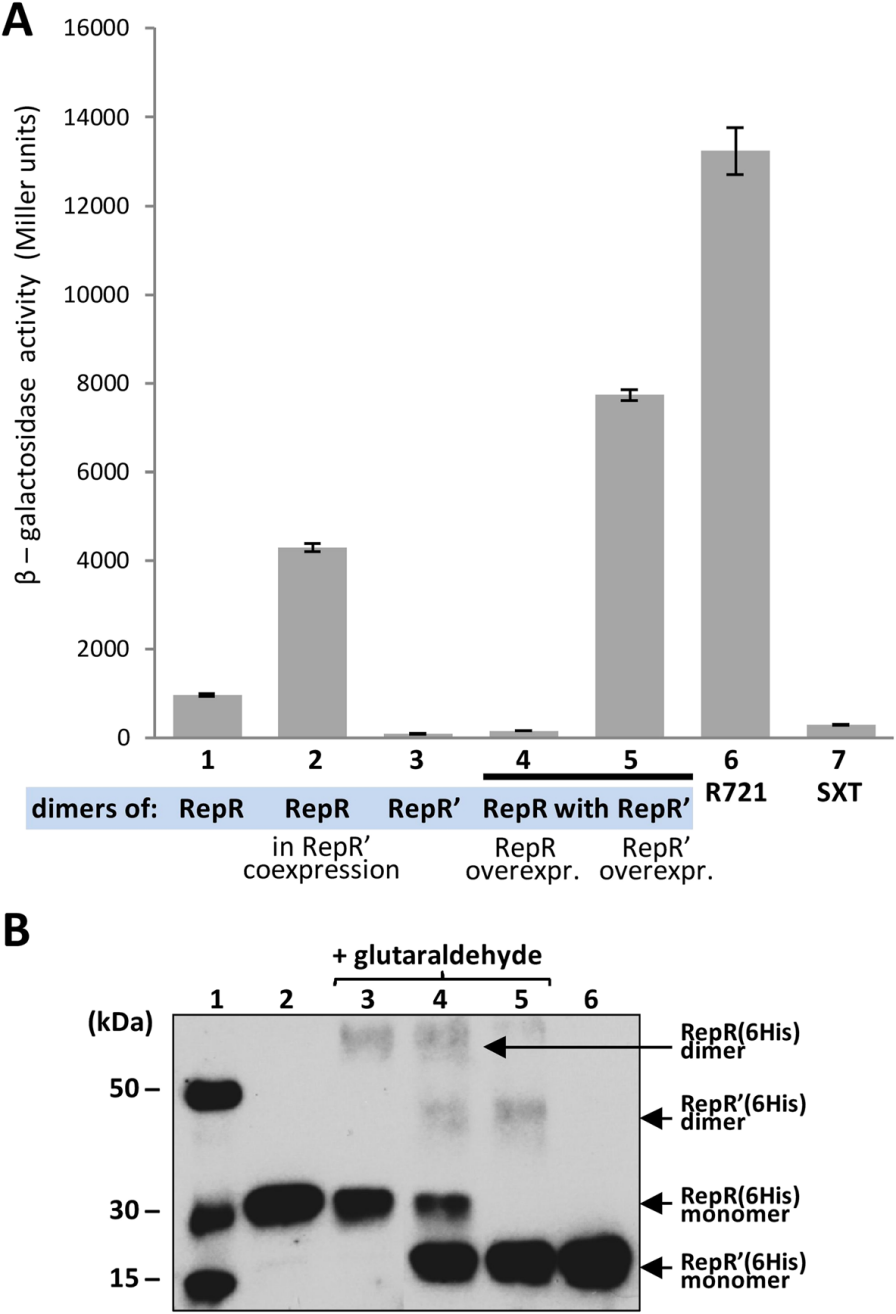
Direct interactions of RepR and RepR’ proteins. **a** In vivo interactions of the RepR and RepR’ proteins determined using a bacterial two-hybrid system. β-Galactosidase activity in *E. coli* R721 strains expressing *N*-terminally fused proteins: (1) RepR alone, (2) RepR co-expressed with wt RepR’, (3) RepR’ alone, (4) RepR plus RepR’ in the presence of RepR overexpression, (5) RepR plus RepR’ in the presence of RepR’ overexpression, (6) plasmid-less *E. coli* strain R721 as a negative control, 7. SXT – positive control system (toxin and antitoxin proteins of the *Vibrio cholerae* SXT element addiction system) [28]. A decrease in β-galactosidase activity relative to the negative control indicates the formation of protein dimers. **b** In vitro interactions of the RepR(6His) and RepR’(6His) proteins determined using glutaraldehyde cross-linking: (1) protein molecular-weight size marker, (2) RepR(6His), (3) RepR(6His) incubated with glutaraldehyde, (4) both RepR(6His) and RepR’(6His) incubated with glutaraldehyde, (5) RepR’(6His) incubated with glutaraldehyde, (6) RepR(6His)

The RepR and RepR’ proteins contain a predicted coiled-coil motif, known to mediate dimer formation in other proteins [27]. To test the ability of RepR and RepR’ to form homo- and/or heterodimers, a bacterial two-hybrid system was used. Different variants of the *repR* gene were cloned into the test vectors pcl_434_ and plc_22_ (Fig. 3a) to produce plasmids able to express recombinant RepR (pcl_434_R and plc_22_R) or RepR’ (pcl_434_R’ and plc_22_R’) proteins. In addition, the wild-type *repR* gene was cloned in both vectors for expression of RepR and RepR’ in its native form, i.e. repressor subunits attached only to the *N*-terminus of RepR (pcl_434_RR’ and plc_22_RR’) (Fig. 3a).

Analysis using these constructs showed that RepR molecules interact with each other to form homodimers (Fig. 3a). The RepR’ protein, like RepR, also forms homodimers. In addition, it was shown that in the presence of RepR’, the level of RepR dimerization is decreased by more than 4-fold compared to the system in which only RepR (wt) is produced.

The formation of Rep heterodimers was further analyzed upon relative overproduction of either RepR or RepR’ (Fig. 3a). The vectors plc_22_ and pcl_434_ both contain the same promoter driving fusion protein transcription, but due to the difference in plasmid copy number, a gene cloned in pcl_22_ (a high-copy-number plasmid with a ColE1-type origin) is more highly expressed than a gene cloned in pcl_434_ (a low-copy-number plasmid with a p15A origin). Preferential formation of heterodimers was observed when RepR was overproduced. When RepR’ was overproduced, the level of heterodimers was significantly lower (Fig. 3a), which suggested that this truncated protein preferentially forms homodimers.

The two- hybrid assays indicated that also heterodimers might be formed although that could not be confirmed by cross-linking experiments (Fig. 3b). We identified RepR (6His) and RepR’(6His) homodimers using the Western blot technique. However, this method does not allow for the detection of RepR (6His) and RepR’(6His) heterodimers.

In short, both RepR and RepR’ are capable of forming homodimers and additional confirmation is necessary to verify if they interact with each other to form heterodimers.

### RepR and RepR’ differ in their DNA-binding properties

**Fig. 4:**
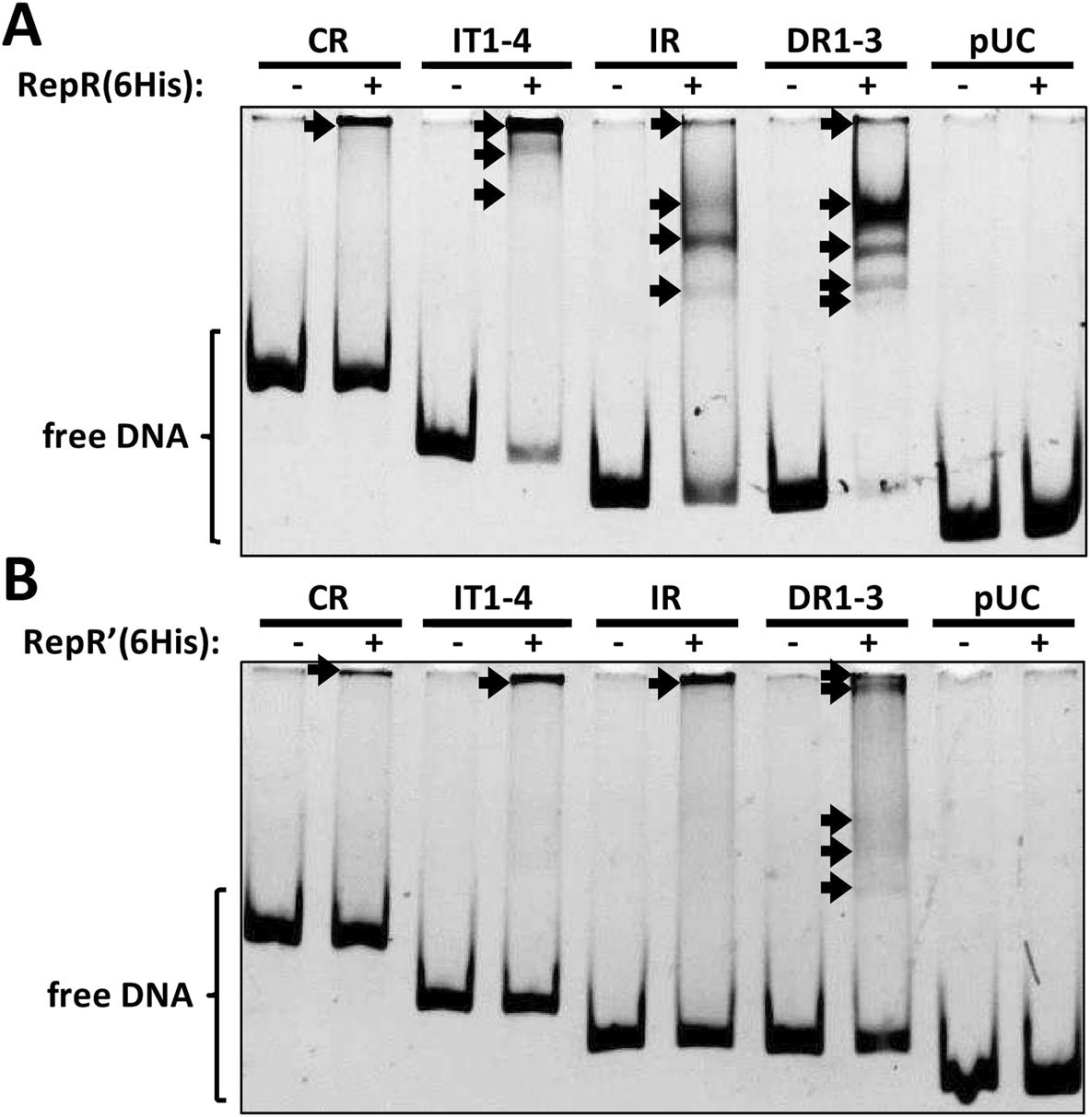
Electrophoretic mobility shift assay (EMSA). **a** and **b** show binding of RepR(6His) and RepR’(6His) to HEX- labeled DNA fragments of pIGRK. Nucleoprotein complexes are marked with arrows. pUC – control DNA fragment from plasmid pUC18

Interactions of RepR and RepR’ with pIGRK DNA were investigated by the electrophoretic mobility shift assay (EMSA). Recombinant RepR (6His) and RepR’(6His) proteins were purified and mixed with one of the four FAM-labeled DNA fragments containing elements indispensable *in cis* for plasmid replication (CR, IT1–4) or potential operator sites of the *repR* promoter region (IR, DR1–3), as well as the control fragment of the pUC18 plasmid (pUC). EMSA assays demonstrated the presence of multiple shifts corresponding to complexes of the RepR (6His) protein with the IR and DR1–3 pIGRK DNA fragments (Fig. 4a). In the case of the CR and IT1–4 fragments, high molecular weight complexes were formed that were unable to enter the gel. No such nucleoprotein complexes were visible in the control reaction (with the pUC fragment). However, we cannot rule out that high molecular weight complexes formation is caused by nonspecific interactions of purified Rep proteins (RepR (6His) and also RepR’(6His) (Fig. 4b)) with low G + C content DNA. The smaller protein, RepR’(6His), could also bind to all four analyzed pIGRK DNAs, although its DNA binding efficiency seemed to be much lower compared to RepR (6His) (Fig. 4b) and only in the case of DR1–3 fragment, shifts corresponding to specific DNA-RepR’(6His) complexes were detected.

### Both RepR and RepR’ autoregulate their own gene expression

The EMSA results indicated that the His-tagged RepR and RepR’ interact with a presumed promoter region of *repR* (Fig. 4a, b), which suggested that both proteins may act as transcriptional regulators. To verify this hypothesis, efforts were made to identify the *repR* promoter (P_*repR*_) and examine the influence of RepR and RepR’ on its activity.

To define the precise location of the promoter, the transcription start site (+ 1) of the *repR* gene was mapped by 5′-RACE to nucleotide position 107 of the pIGRK sequence, i.e. downstream of DR1–3 (Fig. 1a). Notably, the nearby hexameric sequences [5′-TTGACT (15 N)TTAAAA-3′] show significant similarity to the *E. coli* promoter consensus sequence [5′-TTGACA (15-19 N)TATAAT-3′] [29] (Fig. 1a).

A DNA region between the IT1–4 repeats and the start codon of *repR* (containing the predicted P_*repR*_) was then cloned into promoter test vector pRS551, to generate a putative transcriptional fusion with a promoter-less *lacZ* reporter gene (plasmid pRS-rk_12; Fig. 1c). The level of β-galactosidase activity was then measured in a lysate of cells carrying this construct. Unexpectedly, promoter activity was not detected in *E. coli* DH5αΔ*lac* harboring pRS-rk_12 (Fig. 1c). Sequencing of plasmid DNA isolated directly from the bacterial culture used for the enzymatic assay showed that the P_*repR*_ promoter had been inactivated by transposition of insertion sequence IS*1* (Fig. 1c). Analogous transposition events were observed in two other independent experimental approaches (data not shown). It is assumed that the genetic instability of pRS-rk_12 might result from the extremely high activity of the cloned P_*repR*_ promoter deprived of its regulatory elements.

To verify this hypothesis, a strong transcription termination signal, derived from P1 prophage (T*pro*/T*lyz* terminator [30]), was inserted into pRS-rk_12, between the predicted P_*repR*_ and the reporter gene. Diminished transcription from P_*repR*_ stabilized the genetic structure of pRS-rk_9 (no IS*1* insertion mutants were selected). Moreover, despite the presence of a terminator, very high promoter activity was still observed (Fig. 1c).

To investigate the regulatory functions of Rep and RepR’, DNA fragments of pIGRK containing P_*repR*_ along with the wild-type *repR* gene or its mutated forms (producing RepR or RepR’) were cloned in pRS551 (Fig. 1c). Reporter gene expression analysis using these constructs revealed that P_*repR*_ promoter activity was inhibited in the presence of one or both of the proteins RepR and RepR’ (Fig. 1c). This regulatory effect was abolished when DR1–3 and IR were sequentially deleted, confirming their role as operator sequences. Interestingly, the decrease in promoter activity also occurred after the removal of the DR1–3 and IR sequences in the absence of both repressors RepR and RepR’, suggesting a dual function for these regions – as the P_*repR*_ operator and a transcription enhancer. In addition, it was shown that IT1–4 of the pIGRK *origin* also participates in the regulation of P_*repR*_ activity (Fig. 1c).

## Discussion

**Fig. 5:**
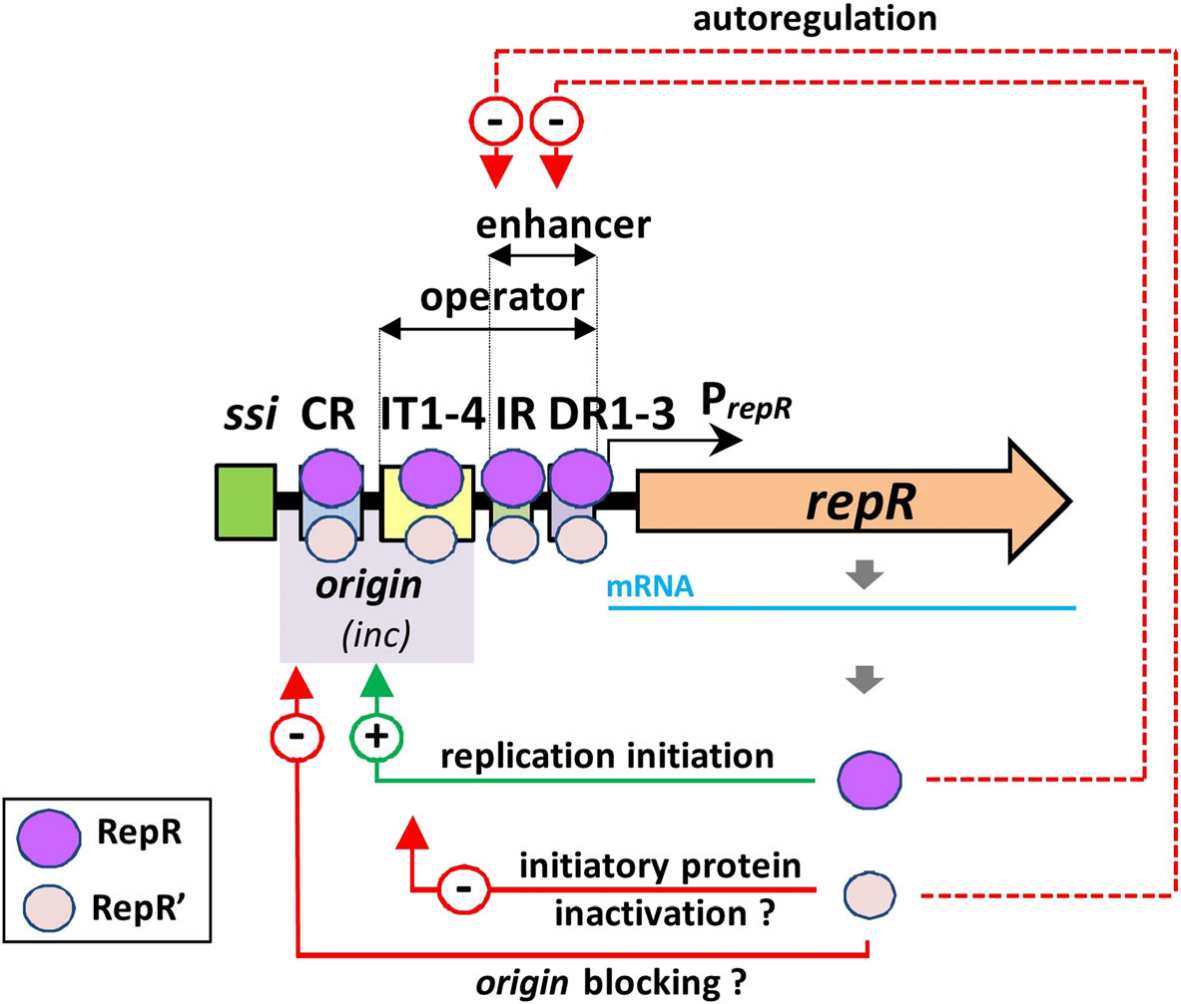
Regulatory role of RepR and RepR’ proteins in the initiation of pIGRK replication. Positive (+) and negative (−) effects, including the proposed mechanisms of RepR’ interference on the initiatory protein functioning, are indicated. The proposed binding sites of RepR and RepR’ within the REP module are indicated. The scheme does not account for how the proteins bind to individual elements of the REP module. The way of RepR and RepR’ binding (as monomers, or dimers) nor its possible cooperative character was considered

This study revealed that a small, cryptic, low-copy-number plasmid pIGRK of *K. pneumoniae*, a member of the pWH126 plasmid family, encodes, in a single *repR locus*, two in-frame proteins – RepR (248 aa) and RepR’ (180 aa). These proteins have several features in common: both (i) contain in silico predicted wHTH (putatively involved in DNA binding and/or protein dimerization) and coiled-coil (another putative dimerization motif) motifs (ii) interact with the same DNA sequences (however with different affinity), (iii) autoregulate their own gene expression, and (iv) are able to form homodimers. Despite these similarities the proteins play distinct roles in pIGRK replication (Fig. 5). RepR activates the *origin* by binding to its iteron-like IT1–4 sequences and CR region, while RepR’ negatively regulates this process. This negative effect may result from binding and blocking the *origin* by RepR’ (monomers or dimers) and/or by the formation of RepR’-RepR heterodimers showing reduced binding affinity for the *origin* (Fig. 4). An increased number of heterodimers may limit the ability of RepR molecules to activate the *origin*.

The aforementioned findings indicate the complexity of the regulatory network controlling pIGRK replication and point to a major role for the RepR and RepR’ proteins (Fig. 5). In their properties, RepR and RepR’ strongly resemble two in-frame proteins – π^35.5^ and π^30.5^ – encoded by the BHR plasmid R6K [31]. The full-length protein, π^35.5^, encoded by a *pir* gene, initiates R6K replication. It interacts with seven iterons as well as with an upstream non-iteron-containing A + T rich region of the origin (*ori*γ) [15]. The shorter protein, π^30.5^, produced from an alternative internal translational START site within the *pir locus* (similarly to RepR’) negatively influences plasmid replication [15]. Furthermore, both π^35.5^ and π^30.5^, like RepR and RepR’, are able to form homodimers, which play different roles in replication [14, 32]. The analogies between the regulatory elements of pIGRK and R6K are clear, although these plasmids are not phylogenetically related, they do not show sequence similarity, and moreover they are likely to use different modes of replication: R6K is a class A theta plasmid [33], while pIGRK is thought to be an RCR replicon [20, 21].

Although in-frame Rep proteins have not been previously reported for RCR plasmids, they were found in several RCR prophages [5]. A good example is phage ΦX174, encoding A and A* proteins, which both possess nuclease activity [34]. Protein A is indispensable for RCR of double-stranded prophage DNA replicative forms (RF) [35]. The biological function of A* is to switch the RF → RF replication of phage genomes into a mode producing single-stranded (SS) DNA molecules (RF → SS) prior to their packaging into capsids [35]. Two in-frame Rep proteins are also produced by filamentous RCR bacteriophages (f1, M13, fd) [5]. The full length protein (pII) is responsible for replication initiation by DNA cleavage, while the shorter protein (pX), acting at a later stage in the phage cycle, promotes the synthesis of single-stranded DNA [5]. Further studies are necessary to determine whether an analogous molecular switch between modes of replication (from RF → RF to RF → SS) occurs in the case of pIGRK, and whether RepR’ is a functional equivalent of the A* or pX proteins.

Besides their involvement in the initiation of pIGRK replication, RepR and RepR’ also act as transcriptional regulators, inhibiting *repR* expression driven by promoter P_*repR*_ (Fig. 5). It was revealed that *repR* is expressed solely from P_*repR*_ and there are no internal promoters engaged in *repR’* expression (Fig. 1, Fig. 2). Rozhon [36] analyzed the regulatory mechanisms of pHW126 and showed that DR1–3 repeats within this plasmid (homologous to those of pIGRK) are operator sequences, where Rep_pHW126_ binds as a transcription inhibitor. In the case of pIGRK we found that three elements influence P_*repR*_ activity – DR1–3, IR and IT1–4 of the *origin*. RepR and RepR’ interact with all these elements (Fig. 4), which, surprisingly, show limited (IT1–4 and IR) or no (DR1–3) reciprocal sequence identity.

Such a complex operator structure may result from the need for precise regulation of a very strong promoter, P_*repR*_. We were unable to clone this promoter in a transcription fusion with *lacZ* gene unless a transcription terminator was inserted between the promoter and reporter gene (Fig. 1). Presumably, the high level of β-galactosidase produced caused toxic effects in *E. coli* cells. The presence of strong *rep* promoters is not unique. This phenomenon was previously reported in several plasmids, both of the *theta* (e.g. R1) and RCR type (e.g. pMV158), and it was suggested that high promoter activity may lead to repopulation of plasmids during colonization of new hosts [37].

This study has provided valuable data on the mechanisms regulating the replication of pIGRK and presumably other members of the pHW126 plasmid family. Nevertheless, more detailed analyses are required to fully understand the molecular basis of RepR and RepR’ function. One future goal is to examine the DNA-binding properties of the individual forms of these proteins, i.e. monomers and dimers, and define the biological role of these interactions. It seems that both RepR and RepR’ are able to recognize different DNA sequences and that RepR’ binds with lower affinity to DNA than RepR (Fig. 4). The results of the EMSA analysis were reproducible and reliable, However, the formation of high molecular weight complexes was confusing. We have to check if these were result of specific or nonspecific DNA binding. Moreover, the presence of in silico-predicted wHTH and coiled-coil motifs, and their involvement in the assembly of the different forms of RepR and RepR’ proteins, should be verified by mutational analyses.

It would also be interesting to know whether in-frame proteins are encoded by other members of the pHW126 plasmid family. In silico analysis has revealed that the *rep* genes of all but one of these plasmids (pRAO1) contain a codon for valine at the position corresponding to the start codon of RepR’. Nonetheless, the putative internal START codon (*gtg*) is only present in the Rep proteins of pIGRK, pHW126 and pSYM8; in other cases valine is encoded by the codon *gtt*. However, the presence of other internal START codons, giving rise to additional regulatory proteins, cannot be excluded [38] and requires further investigation.

It is important to underline that pIGRK was originally isolated from a clinical strain of *Klebsiella pneumoniae,* whereas all the analyses described in this study were performed in *E. coli* host strains. We cannot rule out that the pIGRK plasmid functions differently in these two hosts. However, it should be emphasized that the aforementioned plasmid pSYM8 (representing the pHW126 plasmid family), identified in *E. coli* strain G5, showed 99% nucleotide sequence identity with pIGRK. Therefore, it is likely that the characteristics of pIGRK in both *E. coli* and *K. pneumoniae* are similar. This hypothesis should be verified by future experiments.

This study also revealed that, similar to pHW126 [21], pIGRK is a low-copy number plasmid (< 9 copies/per chromosomal equivalent) [for more details see Additional file 2]. Low-copy-number replicons that have been characterized to date are encoding a set of stabilizing systems that ensure (i) resolution of multimeric plasmid forms into monomers, (ii) precise segregation of plasmid copies into daughter cells during cell division, and (iii) elimination of plasmid-less cells from a bacterial population at the post-segregational level. Plasmid pIGRK lacks these systems, so its high stability depends on as-yet uncharacterized mechanisms, presumably provided by the host cell. Therefore, pIGRK proved to be an intriguing research model, not only for studies on the initiation of DNA replication, but also for the investigation of other basic processes that enable the stable maintenance of plasmid molecules in bacterial cells.

## Conclusions

Regulation of pIGRK replication initiation relies on the activity of two in-frame proteins with antagonistic functions. The coding of two proteins in a single plasmid locus seems to be an uncommon phenomenon, since to the best of our knowledge this was previously reported only in three BHR replicons. However, its occurrence may be underestimated due to the inability to identify OGs encoding in-frame proteins by in silico sequence analysis. This phenomenon is also a novel feature among plasmids of the pHW126 family. pIGRK and pHW126 have highly related REP modules and share several properties, suggesting that their replication proceeds via a common mechanism. pHW126-like plasmids are believed to replicate using a RC mode. However, they display several features that have not been reported for RCR plasmids (e.g. low copy number) or are typical for *theta* replicating plasmids (e.g. autoregularory properties of Rep proteins, a Rep protein with a HTH motif). Further studies on the replication mode of these plasmids are required in order to define the role of the Rep proteins and characterize the replication intermediates.

## Methods

### Bacterial strains, plasmids and culture conditions

Bacterial strains and plasmids used in this study are listed in [Additional file 3: Table S2]. All strains were cultured at 37 °C in lysogeny broth (LB) medium (tryptone 10.0 g/l, yeast extract 5.0 g/l, and NaCl 5.0 g/l; pH 7.2–7.5). When necessary, the medium was supplemented with appropriate antibiotics at the following concentrations: ampicillin (Ap) – 100 μg/ml, kanamycin (Km) – 25 μg/ml [for *E. coli* BL21(DE3)] or 50 μg/ml (for other strains), or chloramphenicol (Cm) – 34 μg/ml. L-Arabinose was added to the medium at a final concentration of 0.2% to induce expression of genes cloned downstream of the *araBAD* promoter in vector pBAD33.

### DNA manipulations

Plasmid DNA was isolated using a Plasmid Mini Isolation Kit (A&A Biotechnology) according to the manufacturer’s instructions. DNA was introduced into bacterial cells by electroporation, using 1-mm gap cuvettes (BTX) and a MicroPulse electroporator (Bio-Rad), as described by Sambrook and Russell [39]. Details of plasmid constructions are presented in [see Additional file 3: Table S2]. Routine DNA manipulations were carried out using standard procedures [39]. All restriction, DNA-modifying enzymes and DNA ligase were supplied by Thermo Fisher Scientific. Amplification of DNA fragments by PCR was performed using Pfu or Taq DNA polymerase (Thermo Fisher Scientific), appropriate primers and template DNAs. Point mutations in the *repR* gene were generated using specific primers and a QuikChange Site-Directed Mutagenesis kit according to the protocol supplied by the manufacturer (Stratagene). All oligonucleotide primers used in this study are listed in [see Additional file 4: Table S3].

### Plasmid stability assay

Segregational stability of pIGRK plasmid derivatives was tested by replica plating following growth under non-selective conditions for approx. Eighty generations, as described previously [40]. The incompatibility characteristics of two plasmids (residing and introduced) were examined by testing the stability of the residing replicon (pRK-1) in the presence of ampicillin in the growth medium (antibiotic selection for introduced pUC18 vector derivatives containing putative determinants of incompatibility of pIGRK).

### Western blot analysis

Protein samples were separated on standard 12% polyacrylamide SDS-PAGE gels. After electrophoresis the gel was incubated in transfer buffer (1x SDS-PAGE buffer, 20% methanol) for 15 min. PVDFD membrane was incubated in methanol for 30 min and briefly washed in transfer buffer. Proteins were transferred from the gel onto the membrane using a Bio-Rad “Trans-blot” run with transfer buffer at 15 V for 60–75 min. The transfer membrane was then bathed overnight in blocking buffer (50 mM Tris-HCl pH 7.5, 150 mM NaCl, 1 mM EDTA pH 8.0, 0.05% Tween 20, 5% milk powder, 1% albumin) at 4 °C. The blocked membrane was incubated with 1:10,000 diluted mouse anti-His tag antibody (EMD Chemicals, 70,796–3) for 1 h at room temperature. Next, the membrane was washed 6 times for 10 min in washing buffer (50 mM Tris-HCl pH 7.5, 150 mM NaCl, 0.1 mM EDTA pH 8.0, 0.05% Tween 20). The membrane was then incubated with 1:10,000 diluted anti-mouse IgG (γ-chain specific) − peroxidase conjugated antibody (Sigma, A3673) for 1 h at room temperature. After a further 6 times 10 min washes, immunoreactive bands on the blot were detected using the ECL Plus Western Blotting Detection System (Amersham), according to the manufacturer’s instructions.

### Overexpression and purification of 6His-tagged Rep proteins

Rep proteins were overexpressed and purified using a method described by Rozhon et al. [21] with some modifications. The *repR* gene variants of pIGRK were cloned in expression vector pET28b + resulting in pET-*repR*_6HV69V_ (for RepR (6His) overexpression) and pET-*repR*_6HΔN_ (for RepR’(6His) overexpression) listed in [Additional file 3: Table S2]. *E. coli* BL21(DE3) strains harboring each construct were cultured overnight in LB medium supplemented with kanamycin at 37 °C with shaking (180 rpm). For protein overexpression, 8 ml of the overnight cultures were added to 1000 ml of fresh LB + kanamycin medium, and incubation was continued at 28 °C with shaking (180 rpm). When the culture had reached an OD_600_ of between 0.35 and 0.4, isopropyl β-D-1-thiogalactopyranoside (IPTG) was added to a final concentration of 0.4 mM to induce the expression of the 6His-tagged proteins. The cultures were then incubated further to an OD_600_ 1.0 under the same conditions. The cells were collected by centrifugation (15 min, 6500 xg, 4 °C) and resuspended in 15 ml of lysis buffer: 50 mM sodium phosphate pH 8.0, 300 mM NaCl, 10 mM imidazole, 0.1% triton X-100, and 300 μl of 100 mg/ml lysozyme, supplemented with 1 mM PMSF and Protease Inhibitor Cocktail (Sigma). After holding on ice for 15 min the cells were disrupted by sonication and the obtained lysates were centrifuged (15 min, 22,000 xg, 4 °C) to pellet cell debris. All subsequent steps were performed at 4 °C. The cleared lysates were incubated with 0.25 ml of Ni-NTA beads (Qiagen) for 30 min, with gentle shaking. The Ni-NTA resin was then given a series of washes: (i) twice with 8 ml of W1 buffer (50 mM sodium phosphate pH 8.0, 300 mM NaCl, 20 mM imidazole), (ii) once with 4 ml of W2 buffer (50 mM sodium phosphate pH 8.0, 2 M NaCl, 20 mM imidazole), and finally (iii) three times with 8 ml of W1 buffer. The 6His-tagged proteins were finally eluted with 0.4 ml of elution buffer (50 mM sodium phosphate pH 8.0, 300 mM NaCl, 150 mM imidazole). The concentration of the purified recombinant proteins was estimated using the Bradford dye-binding method. All proteins were analyzed by SDS-PAGE and MALDI-TOF mass spectrometry to confirm their identity. Protein aliquots were frozen in liquid nitrogen and stored at − 70 °C.

### Electrophoretic mobility shift assay

#### Obtaining fluorescein (FAM)-labeled DNA fragments

The following elements of the REP module of pIGRK were cloned in vector pUC18: (i) CR (pUC-RK4_2), (ii) IT1–4 (pUC-RK-4_1), (iii) IR (pUC-RK_21) and (iv) DR1–3 (pUC-RK_22) (Additional file 3: Table S2). Using these plasmid constructs as templates the cloned DNA fragments were amplified by PCR with “universal” M13 forward and reverse primers – M13pUCf and FAM-labeled M13pUCrFAM (oligos 42 and 43 in [Additional file 4: Table S3]), and subsequently purified using a Clean-Up kit (A&A Biotechnology). The same primer pair was used for the amplification of a 136-bp DNA fragment of pUC18, which served as a negative control.

#### DNA binding assay

Binding reactions of the total volume of 18 μl, containing 2 μl of 10x TEKEM buffer [41] [200 mM Tris-HCl (pH 8.0), 1000 mM KCl, 10 mM EDTA, 3 μg of poly-dIC competitor, 40 μg of BSA (bovine serum albumin)], were incubated for 5 min at room temperature with 1 μl of: (i) RepR(6His) (approx. 500 ng - 16 pmol) or (ii) RepR’(6His) (approx. 350 ng - 16 pmol) or (iii) H_2_O (control reaction). The approximate protein amounts were calculated based on the Bradford dye-binding method and SDS-PAGE analysis [Additional file 5]. Subsequently, 0.25 pmol of FAM-labeled DNA fragments were added to the final volume of 20 μl. After 30 min incubation at 25 °C, the reactions were gently mixed with 6 μl of 50% glycerol and loaded on a standard 6% polyacrylamide gel cast with TBE. Protein-DNA complexes were then separated by electrophoresis in 1 x TBE buffer at 10 V/cm at 10 °C and DNA fragments were visualized using Imager 600 (Amersham).

### Glutaraldehyde cross-linking

Glutaraldehyde cross-linking experiments were performed according to the procedure described by Rozhon [24]. The formation of Rep proteins dimers was investigated by incubation of approx. 100 ng of: (i) RepR(6His), (ii) RepR’(6His) or (iii) RepR(6His) and RepR’(6His) with 100 μM of glutaraldehyde. Protein samples were separated by SDS-PAGE and Rep proteins were identified by Western blotting.

### Determination of the plasmid copy number

Plasmid copy number was determined by QPCR (Quantitative Polymerase Chain Reaction) as described previously [42]. DNA primers specific to (i) the kanamycin resistance gene (*kan*) (each of the tested plasmids carried a single copy of this gene) – oligos 55 and 56, [Additional file 4: Table S3] and (ii) the D-1-deoxyxylulose 5-phosphate synthase gene (*dxs*) [43] (a reference single-copy gene present in the *E. coli* chromosome) – oligos 57 and 58, [Additional file 4: Table S3] were used.

#### Preparation of template DNA for QPCR

The absolute copy number of the pRK-1 plasmid was determined by QPCR, performed in triplicate (three independent clones were analyzed) [see Additional file 2]. To determine the effect of (i) *ssi* sequence deletion, or (ii) changes in RepR or RepR’ quantities on the pIGRK derivatives copy number, material from a single bacterial colony was used for analysis. Total DNA was extracted from each of the cultures during the exponential growth phase, which was determined by periodic measurements of the optical density (OD_600_), when the parameter value was approx. 0.6. The extraction was performed using QIAamp DNA Mini Kit (Qiagen), following the method for bacterial cells described in the manufacturer’s instructions (for preparing a template directly from a culture broth). Additional template preparation methods were used for determination of absolute pRK-1 copy number. Bacterial pellets from liquid cultures were suspended in water and boiled. Cell lysates were centrifuged and obtained supernatants were added to QPCR reactions. Alternatively, bacterial pellets (after rinsing with water) were directly added to QPCR reactions.

#### Construction of the standard curve for plasmid copy number determination

The *dxs* gene fragment from *E. coli* chromosome was amplified by conventional PCR using specific primers. The PCR product was purified using Gel Out Kit (A&A Biotechnology) and cloned into the pDrive cloning vector (Qiagen) [25]. The resulting plasmid pDrivedxs contained two separate sequences: (i) the *dxs* gene fragment and (ii) the *kan* gene (pDrive vector contains a *kan* sequence identical with that present in pIGRK derivatives). Thus, the pDrivedxs plasmid could be detected by using either *kan* -or *dxs*-set. A series of 10-fold dilutions of this plasmid, ranging from 1.6 × 10^2^ to 1.6 × 10^7^ copies/μl, were used to construct standard curves for both *kan* and *dxs*. The concentration of the plasmid DNA was measured spectrophotometrically and the corresponding copy number was calculated using the equation described by Lee et al. [2006].

#### Real-time QPCR

Real-time QPCR amplification and analysis were performed using a Stratagene M × 3000P™ real-time PCR instrument. The threshold cycle (CT) was determined by the “Fit Points Method” in the instrument software. The real-time QPCR mixture of 20 μl was prepared using Brilliant III Ultra-Fast SYBR Green QPCR Master Mix (Applied Biosystems): 6.5 μl PCR-grade water, 0.5 μl of each primer, 10 μl of the reaction mix 2 × solution, and 2.5 μl of the template DNA. The cycling protocol was: 3 min at 95 °C and 30 cycles of 20 s at 95 °C, 20 s at 60 °C. Experiments were carried out in triplicate (three QPCR reactions for each DNA isolate) and the results are reported as mean values. The fluorescence signal was measured at the end of each extension step. Following amplification, a melting curve analysis with the temperature gradient of 0.1 °C/s from 60 to 95 °C confirmed that only the specific products were amplified.

#### Quantification in QPCR

Quantification was performed using the standard curves constructed for both *kan* and *dxs*. The copy numbers of *kan* and *dxs* in the *E. coli* total DNA samples were determined from the corresponding standard curves, using the CT values. The plasmid copy number of pIGRK derivatives was then calculated by dividing the copy number of *kan* by the copy number of *dxs*. As *kan* and *dxs* are single-copy genes of pIGRK and *E. coli* chromosomal DNA, respectively, the ratio of *kan* to *dxs* is equal to the plasmid copy number of the pIGRK *kan* derivatives.

### 5′-RACE for determination of the *repR* transcriptional start site

#### RNA isolation

An overnight bacterial culture was diluted 1:50 in fresh medium and cultivated for a further 3 h. The cells were harvested by centrifugation and total RNA was isolated from the pellet using a Total RNA Mini Plus kit (A&A Biotechnology) and DNase treated using a Clean-Up RNA Concentrator kit (A&A Biotechnology). The quality and concentration of the isolated RNA was evaluated using a Picodrop Microliter UV/Vis Spectrophotometer and standard formaldehyde agarose gel electrophoresis. RNA was stored at − 20 °C.

#### 5′-RACE (rapid amplification of cDNA ends)

To determine the *repR* transcription start site a 2nd Generation 5′/3′ RACE Kit (Roche) was used according to the manufacturer’s instructions with some modifications. Since the pIGRK sequence contains a high level of A + T pairs, Poly [C] tailing of cDNA was applied instead of Poly [A] tailing. Consequently the Oligo dT-anchor primer (included in the kit) was replaced by an Oligo dG-anchor primer (oligo 32, [Additional file 4: Table S3]), during the primary PCR. For cDNA synthesis, 1 μg of total RNA was used with the gene-specific primer RACESP1 (oligo 39, [Additional file 4: Table S3]). Two separate PCR amplifications were performed using dG-tailed cDNA as the template, the first with an Oligo dG-anchor primer (included in the kit) and gene-specific primer SP2RACE (oligo 33, [Additional file 4: Table S3]). The products of these primary reactions were then used as the template in a secondary PCR, with PCR anchor primer (included in the kit) and gene-specific primer SP3RACE (oligo 34, [Additional file 4: Table S3]). The amplified DNA fragment was visualized by agarose gel electrophoresis, then purified using a Gel-Out kit (A&A Biotechnology) and sequenced.

### In vivo Rep proteins interactions assay

Rep proteins interactions were analyzed using bacterial two-hybrid system constructed by Di Lallo and co-workers [26]. This system uses the *E. coli* R721 test strain carrying chromosomally encoded *lacZ* reporter gene and two expression vectors (both enabling recombinant proteins production from IPTG inducted *lacZ* promoter). The *lacZ* promoter present in *E. coli* R721 chromosome contains a hybrid operator with binding sites for (i) phage P434 repressor (encoded in the test vector pcl_434_) and (ii) phage P22 repressor (encoded in the test vector pcl_22_). If the analyzed proteins, overproduced in translational fusions with the repressor subunits, interact directly with each other, an active hybrid repressor is formed and R721 *lacZ* promoter activity is inhibited. The measure of dimer formation in this system is therefore the decrease in β-galactosidase activity in comparison with the control strain R721 [26]. Descriptions of the constructed recombinant pcl_22_ and pcl_434_ vectors are listed in [Additional file 3: Table S2].

### β-Galactosidase assay

β-Galactosidase activity assays were performed according to a method described by Thibodeau and co-workers [44], with slight modifications. *E. coli* strains were cultivated overnight in liquid LB medium supplemented with suitable antibiotics. These cultures were diluted 1:50 in fresh medium and cultivated for a further 2 h. For the Rep protein interaction assay (bacterial two-hybrid system) the LB medium was supplemented with IPTG to a final concentration of 0.1 mM according to Di Lallo and co-workers [45]. The OD_595_ of the bacterial cultures was then measured and eight replicates of 80 μl of each culture were transferred to a 96-well microtiter plate. To cause cell lysis, 20 μl of PopCulture lysis buffer (Merck Millipore) were added to each well, and the mixtures incubated for 15 min at room temperature. Twenty microliters of the lysates were then added to wells of another 96-well plate containing 130 μl of Z buffer. To initiate the enzymatic reaction, 30 μl of the β-galactosidase substrate ONPG (4 mg/ml) (Sigma) were added to each well. The plate was then placed in a TECAN plate reader (Tecan Group Ltd) and incubated at 28 °C as the OD_415_ was measured at 30 s intervals.

### Bioinformatic analyses

DNA or protein sequences were aligned using BLAST (https://blast.ncbi.nlm.nih.gov/Blast.cgi) [46]. Protein secondary structures were predicted with YASPIN Secondary Structure Prediction (https://ibi.vu.nl/programs/yaspin) [47]. Molecular masses and isoelectric points of proteins were predicted using Compute pI/Mw from the Expasy server (https:// web.expasy.org) [48].

## Supplementary information

**Additional file 1: Figure S1.** Mutational analysis of the pIGRK REP module. Contains a detailed description of mutational analysis performed, the results obtained and a schematic representation of the constructed plasmids. (rtf with Figure S1 in jpg format).

**Additional file 2: Table S1.** Determination of pRK-1 plasmid copy number (PCN) in *E. coli* DH5α strain. Plasmid copy number was defined for three independent clones (1–3). For each of DNA isolate three QPCR reactions were performer (the table contains average values). **Figure S2.** Raw data from construction of standard curves and QPCR of total DNA preparates from *E. coli* DH5α clones harboring pRK-1. DNA isolated using QIAamp DNA Mini Kit (Qiagen). **Figure S3.** Raw data from construction of standard curves and QPCR of total DNA preparates from *E. coli* DH5α clones harboring pRK-1. DNA isolated by thermal lysis. Cells were suspended in water, boiled and centrifuged (supernatant used as a template). **Figure S4.** Raw data from construction of standard curves and QPCR of total DNA preparates from *E. coli* DH5α clones harboring pRK-1. Washed cells added directly to the PCR reaction.

**Additional file 3: Table S2.** Bacterial strains, plasmids and genetic cassettes used in this study (rtf).

**Additional file 4: Table S3.** Oligonucleotides used in this study (rtf).

**Additional file 5: Figure S5.** SDS-PAGE analysis of over-expression and purification of RepR(6His) and RepR’(6His) (rtf with Figure S5 in jpg format).

## Data Availability

The datasets used and/or analysed during the current study (which are not included in this published article or its supplementary information files) are available from the corresponding author on reasonable request.
