# Supplementary material for: Molecular dissection of the replication system of plasmid pIGRK encoding two in-frame Rep proteins with antagonistic functions
Source: BMC Microbiol. 2019 Nov 13;19:254. doi: 10.1186/s12866-019-1595-3 (PMC6854812; doi:10.1186/s12866-019-1595-3)
Supplement: Supplementary file 1 — Additional file 1: Figure S1. Mutational analysis of the pIGRK REP module. Contains a detailed description of mutational analysis performed, the results obtained and a schematic representation of the constructed plasmids. (rtf with Figure S1 in jpg format). [file 12866_2019_1595_MOESM1_ESM.zip › Additional file 1.rtf]

Additional file 1


Fig. S1 Determination of the minimal pIGRK replicon. A Genetic organization of pIGRK. Lines represent DNA fragments of pIGRK used to determine the minimal replicon. The insertion sites for the kanamycin resistance cassette (KM) and the replication origin of R6K (oriã) are indicated by black triangles. The ability (+) or inability (-) of the constructed plasmids to replicate in E. coli DH5á or E. coli DH5á (repR) harboring pUC-repR_1 is indicated (nd – not determined). B Comparison of plasmid pRK-1 and its variant pRK-1_1 lacking the ssi sequence. Plasmid copy number and stability. Electrophoretic separation of purified plasmid DNA on a 1% agarose gel showing the different forms. C Schematic representation of pRK-1_5ã (repR deficient pIGRK derivative) and  pUC-repR_1 (source of RepR and RepR') plasmids constructed for trans-activation of replication origin. In pUC-repR_1 ampicillin resistance cassette (AP) and replication origin (ori pMB1) were indicated.
