# Supplementary figures and images for: Molecular dissection of the replication system of plasmid pIGRK encoding two in-frame Rep proteins with antagonistic functions
Source: BMC Microbiol. 2019 Nov 13;19:254. doi: 10.1186/s12866-019-1595-3 (PMC6854812; doi:10.1186/s12866-019-1595-3)

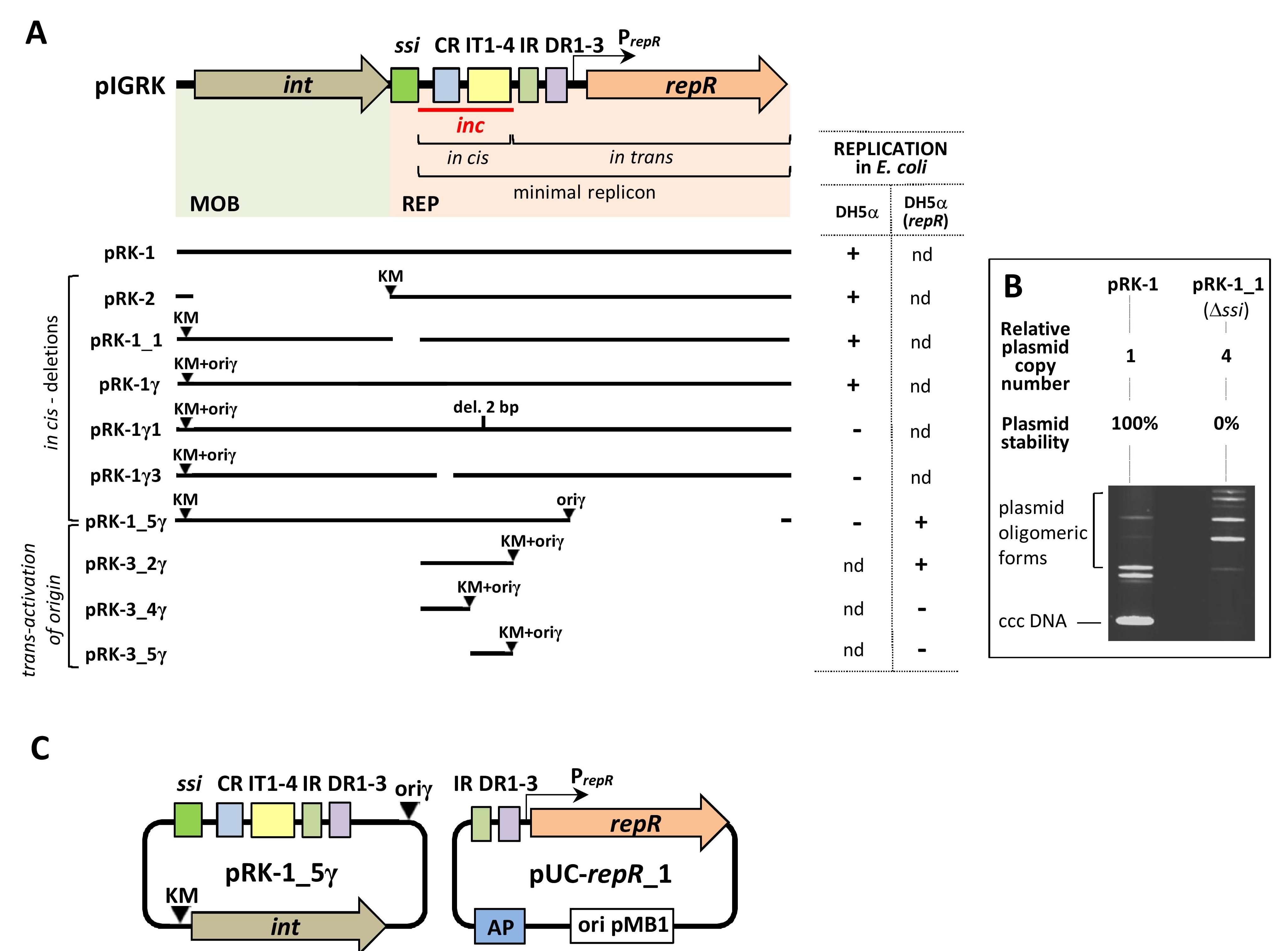

Supplement: Supplementary file 1 — Additional file 1: Figure S1. Mutational analysis of the pIGRK REP module. Contains a detailed description of mutational analysis performed, the results obtained and a schematic representation of the constructed plasmids. (rtf with Figure S1 in jpg format). [file 12866_2019_1595_MOESM1_ESM.zip › Fig S1 from Additional file 1.jpg]

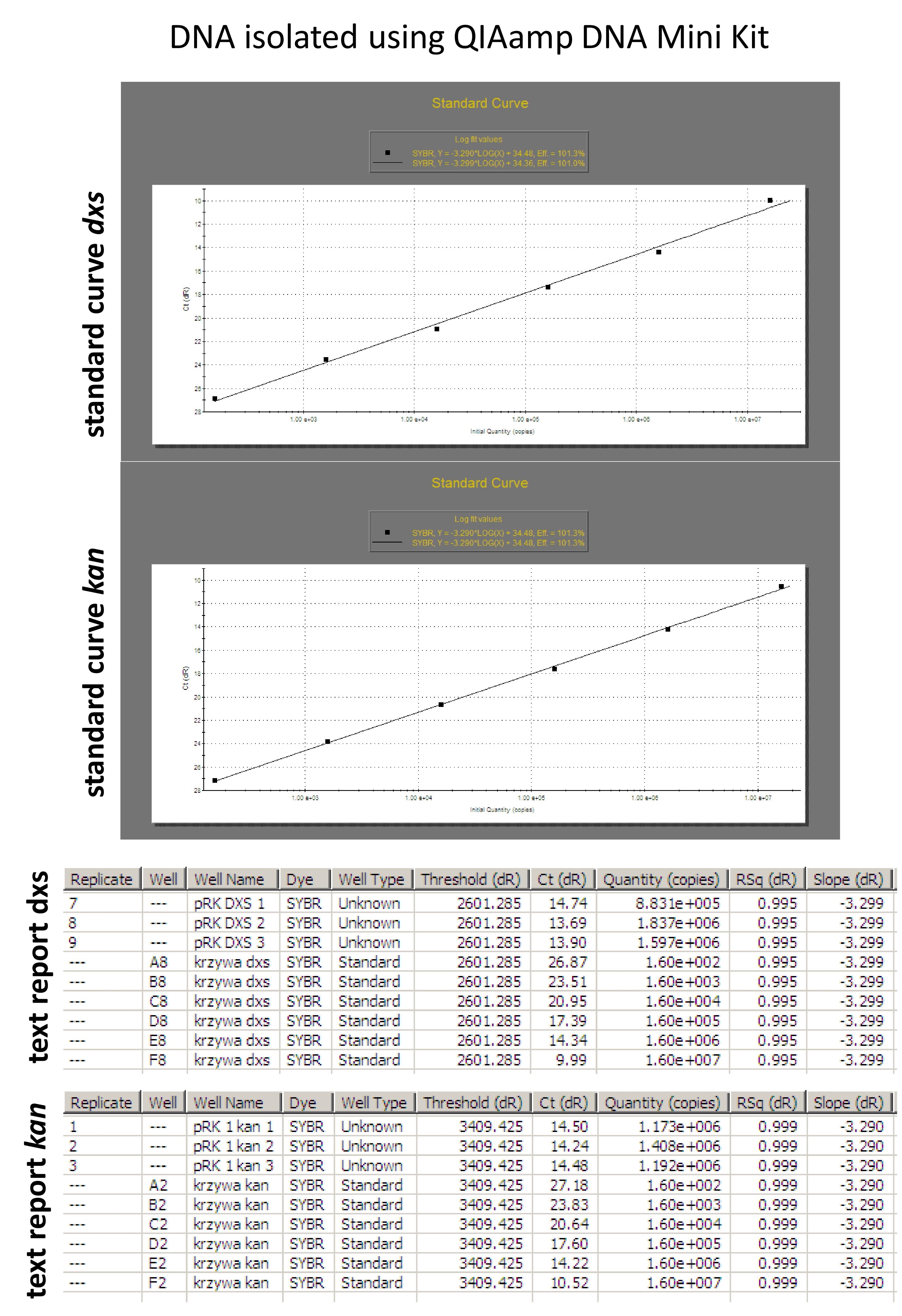

Supplement: Supplementary file 2 — Additional file 2: Table S1. Determination of pRK-1 plasmid copy number (PCN) in E. coli DH5α strain. Plasmid copy number was defined for three independent clones (1–3). For each of DNA isolate three QPCR reactions were performer (the table contains average values). Figure S2. Raw data from construction of standard curves and QPCR of total DNA preparates from E. coli DH5α clones harboring pRK-1. DNA isolated using QIAamp DNA Mini Kit (Qiagen). Figure S3. Raw data from construction of standard curves and QPCR of total DNA preparates from E. coli DH5α clones harboring pRK-1. DNA isolated by thermal lysis. Cells were suspended in water, boiled and centrifuged (supernatant used as a template). Figure S4. Raw data from construction of standard curves and QPCR of total DNA preparates from E. coli DH5α clones harboring pRK-1. Washed cells added directly to the PCR reaction. [file 12866_2019_1595_MOESM2_ESM.zip › Fig S2 from Additional file 2.jpg]

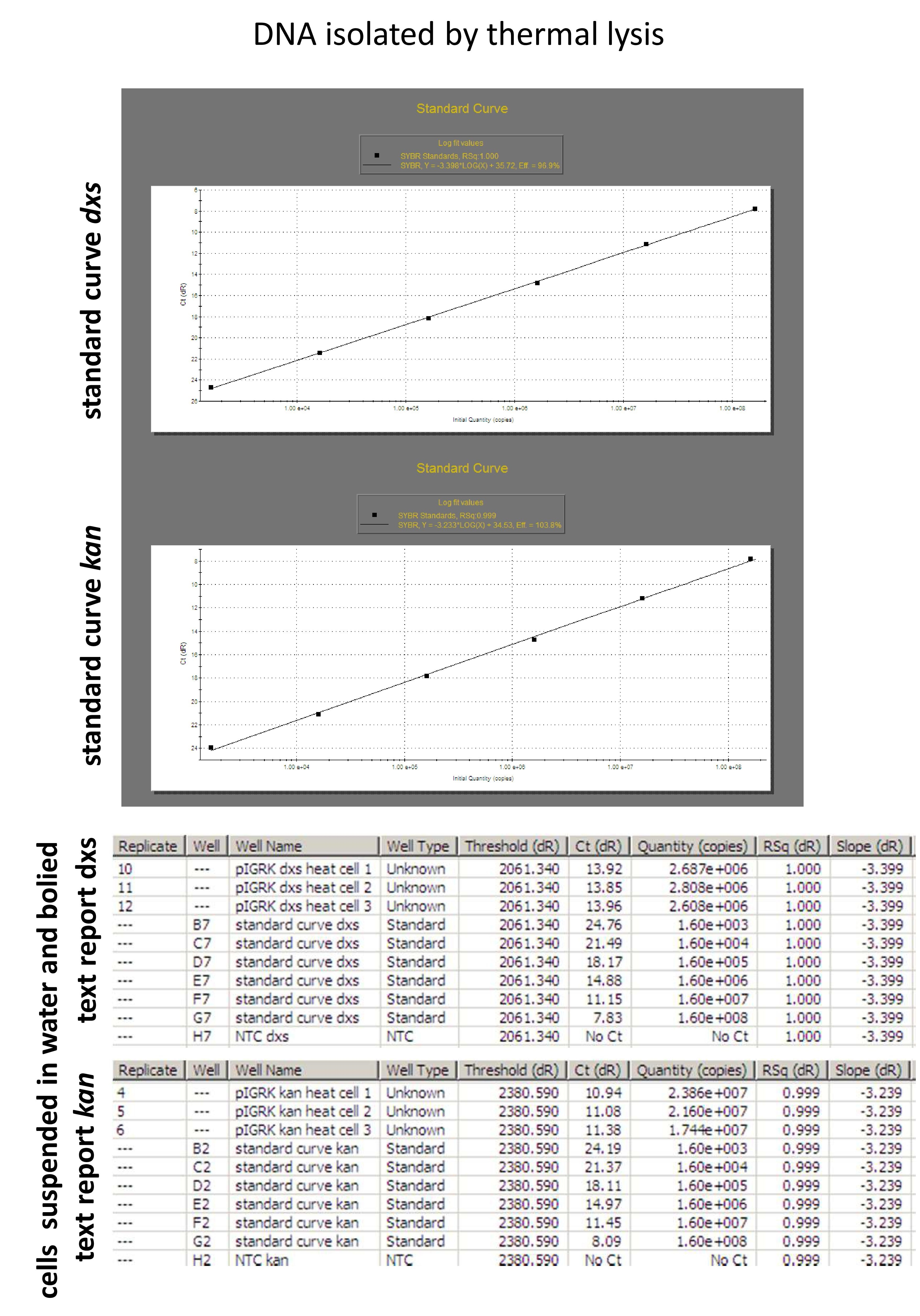

Supplement: Supplementary file 2 — Additional file 2: Table S1. Determination of pRK-1 plasmid copy number (PCN) in E. coli DH5α strain. Plasmid copy number was defined for three independent clones (1–3). For each of DNA isolate three QPCR reactions were performer (the table contains average values). Figure S2. Raw data from construction of standard curves and QPCR of total DNA preparates from E. coli DH5α clones harboring pRK-1. DNA isolated using QIAamp DNA Mini Kit (Qiagen). Figure S3. Raw data from construction of standard curves and QPCR of total DNA preparates from E. coli DH5α clones harboring pRK-1. DNA isolated by thermal lysis. Cells were suspended in water, boiled and centrifuged (supernatant used as a template). Figure S4. Raw data from construction of standard curves and QPCR of total DNA preparates from E. coli DH5α clones harboring pRK-1. Washed cells added directly to the PCR reaction. [file 12866_2019_1595_MOESM2_ESM.zip › Fig S3 from Additional file 2.jpg]

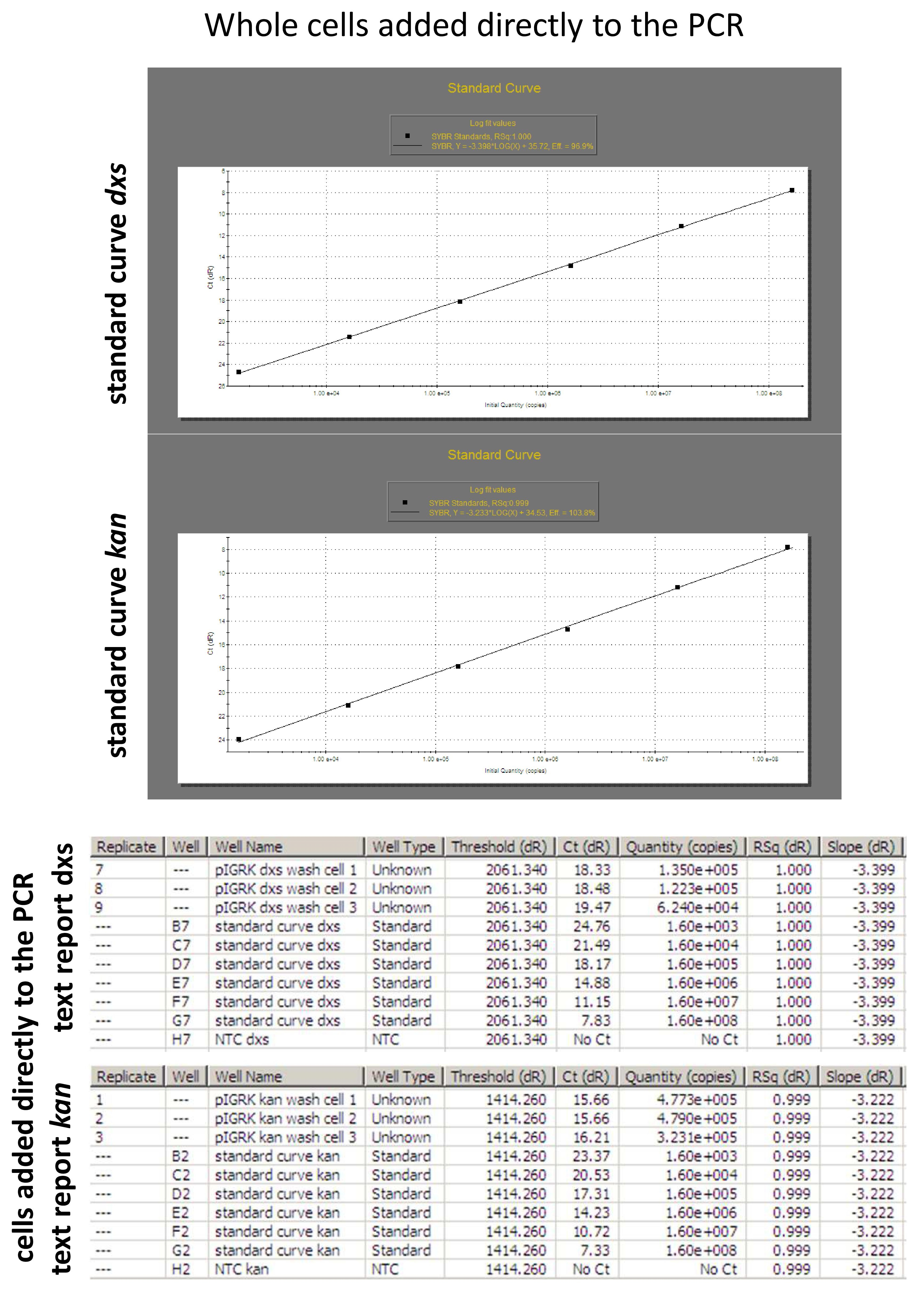

Supplement: Supplementary file 2 — Additional file 2: Table S1. Determination of pRK-1 plasmid copy number (PCN) in E. coli DH5α strain. Plasmid copy number was defined for three independent clones (1–3). For each of DNA isolate three QPCR reactions were performer (the table contains average values). Figure S2. Raw data from construction of standard curves and QPCR of total DNA preparates from E. coli DH5α clones harboring pRK-1. DNA isolated using QIAamp DNA Mini Kit (Qiagen). Figure S3. Raw data from construction of standard curves and QPCR of total DNA preparates from E. coli DH5α clones harboring pRK-1. DNA isolated by thermal lysis. Cells were suspended in water, boiled and centrifuged (supernatant used as a template). Figure S4. Raw data from construction of standard curves and QPCR of total DNA preparates from E. coli DH5α clones harboring pRK-1. Washed cells added directly to the PCR reaction. [file 12866_2019_1595_MOESM2_ESM.zip › Fig S4 from Additional file 2.jpg]

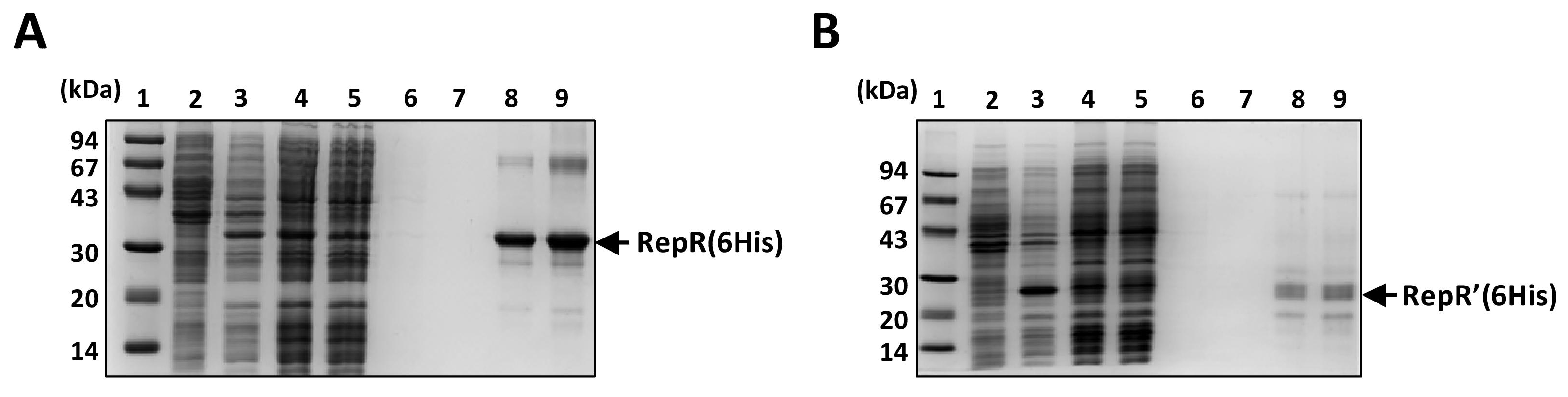

Supplement: Supplementary file 5 — Additional file 5: Figure S5. SDS-PAGE analysis of over-expression and purification of RepR(6His) and RepR’(6His) (rtf with Figure S5 in jpg format). [file 12866_2019_1595_MOESM5_ESM.zip › Fig. S5 from Additional file 5.jpg]
