# Supplementary material for: Molecular dissection of the replication system of plasmid pIGRK encoding two in-frame Rep proteins with antagonistic functions
Source: BMC Microbiol. 2019 Nov 13;19:254. doi: 10.1186/s12866-019-1595-3 (PMC6854812; doi:10.1186/s12866-019-1595-3)
Supplement: Supplementary file 2 — Additional file 2: Table S1. Determination of pRK-1 plasmid copy number (PCN) in E. coli DH5α strain. Plasmid copy number was defined for three independent clones (1–3). For each of DNA isolate three QPCR reactions were performer (the table contains average values). Figure S2. Raw data from construction of standard curves and QPCR of total DNA preparates from E. coli DH5α clones harboring pRK-1. DNA isolated using QIAamp DNA Mini Kit (Qiagen). Figure S3. Raw data from construction of standard curves and QPCR of total DNA preparates from E. coli DH5α clones harboring pRK-1. DNA isolated by thermal lysis. Cells were suspended in water, boiled and centrifuged (supernatant used as a template). Figure S4. Raw data from construction of standard curves and QPCR of total DNA preparates from E. coli DH5α clones harboring pRK-1. Washed cells added directly to the PCR reaction. [file 12866_2019_1595_MOESM2_ESM.zip › Additional file 2.rtf]

Additional file 2
DNA isolation	Replicate
	CT
kan	CT
dxs	Quantity (copies)
kan	Quantity (copies)
dxs	PCN	


QIAamp DNA Mini Kit (Qiagen)	1
	14.50	14.74	1.173 x 10^6^	8.831 x 10^5^	1.3	
	2
	14.24	13.69	1.408 x 10^6^	1.837 x 10^6^	0.8	
	3
	14.48	13.90	1.196 x 10^6^	1.597 x 10^6^	0.8	
thermal lysis of cells suspended in water	1
	15.66	18.33	4.773 x 10^5^	1.350 x 10^5^	3.5	
	2
	15.66	18.48	4.790 x 10^5^	1.223 x 10^5^	3.9	
	3
	16.21	19.76	3.231 x 10^7^	6.240 x 10^6^	5.2	
washed cells added directly to the PCR reaction	1
	10.94	13.92	2.386 x 10^7^	2.687 x 10^6^	8.8	
	2
	11.80	13.85	2.160 x 10^7^	2.808 x 10^6^	7.7	
	3
	11.38	13.96	1.744 x 10^7^	2.608 x 10^6^	6.7	
Table S1 Determination of pRK-1 plasmid copy numer (PCN) in E. coli DH5á strain. Plasmid copy numer was defined for three independent clones (1-3). For each of DNA isolate three QPCR reactions were performer (the table contains average values).


Fig. S2 Raw data from construction of standard curves and QPCR of total DNA preparates from E. coli DH5á clones harboring pRK-1. DNA isolated using QIAamp DNA Mini Kit (Qiagen).
Fig. S3 Raw data from construction of standard curves and QPCR of total DNA preparates from E. coli DH5á clones harboring pRK-1. DNA isolated by thermal lysis. Cells were suspended in water, boiled and centrifuged (supernatant used as a template). 
Fig. S4 Raw data from construction of standard curves and QPCR of total DNA preparates from E. coli DH5á clones harboring pRK-1. Washed cells added directly to the PCR reaction.
