# Supplementary material for: Molecular dissection of the replication system of plasmid pIGRK encoding two in-frame Rep proteins with antagonistic functions
Source: BMC Microbiol. 2019 Nov 13;19:254. doi: 10.1186/s12866-019-1595-3 (PMC6854812; doi:10.1186/s12866-019-1595-3)
Supplement: Supplementary file 3 — Additional file 3: Table S2. Bacterial strains, plasmids and genetic cassettes used in this study (rtf). [file 12866_2019_1595_MOESM3_ESM.rtf]

Additional file 3 

Table S2 Bacterial strains, plasmids and genetic cassettes used in this study.	
Bacterial strain	Description		
E. coli DH5á	F-endA1 glnV44  thi-1 recA1 relA1 gyrA96 deoR nupG Ö80d lacZÄM15 Ä(lacZYA-argF)U169, hsdR17 (rK- mK+), ë	Invitrogen	
E. coli DH5áÄlac	deoR thi1 relA1 supE44 endA1 gyrA96 recA1 hsdR17 Ä(argF lac)U169 Nalr	M. Yarmolinski*	
E. coli R721	supE thy ∆(lac–proAB) F' [proAB+ lacIq lacZ∆M15] 71/18 glpT::O-P434/P22 lacZ	[1]	
E. coli BL21(DE3)	F- ompT gal dcm lon hsdSB (rB- mB-) ë(DE3) pLysS(CmR)	Invitrogen	
E. coli MC1061	F- Ä(ara-leu)7697 [araD139]B/r Ä(codB-lacI)3 galK16 galE15 ë- e14- mcrA0 relA1 rpsL150(strR) spoT1 mcrB1 hsdR2(r-m+)	[2]	
E. coli DH5áëpir
	sup E44 ÄlacU169(ÖlacZÄM15) recA1 endA1 hsdR17 thi-1 gyrA96 relA1 ëpir phage lysogen	[3]	
Plasmid	Description		
pIGRK	2348 bp cryptic plasmid isolated from Klebsiella pneumoniae 287-w	[4]	
pUC18	Apr, ori pMB1, lacZ (M13mp18/19), cloning vector	Qiagen	
pABB19	Apr; ori pMB1, cloning vector with Tpro/Tlyz (T) transcriptional terminator from bacteriophage P1, used for cloning of DNA fragments with T sequence 	[5]	
  pET28b+	Kmr, ori pBR, ori F1, plasmid vector for 6His tagged proteins overexpression, used for Rep proteins identification and purification	Invitrogen	
pcI434	Kmr, ori p15A, pACYC177 derivative coding N-terminal part of P434 phage repressor, used in bacterial two hybrid system
	[1]	
pcI22	Apr, ori ColE1, pC132 derivative coding N-terminal part of P22 phage repressor, used in bacterial two hybrid system	[1]	
pcI434SXT	pcl434 derivative coding protein from SXT N15 prophage	[6]	
pcI22SXT	pcl22 derivative coding protein from SXT N15 prophage	[6]	
pRS551	Apr, Kmr , ori pBR, test vector coding promoter less lacZ reporter gene, used for determination of PrepR promoter activity 	[7]	
pDrivedxs	pDrive with cloned fragment of dxs E. coli gene	[8]	
pBAD33	Cmr, ori p15A, PBAD-araC, expression vector used for arabinose inducted Rep proteins in trans delivery	[9]	
pDS132	Cmr ori R6K, mobRP4, sacB, used for oriã cassette construction	[10]	
pRK-1	pIGRK DNA was PCR amplified using oligo 1 and 2 (Table S2), BamHI digested
and ligated with BglII digested KM cassette (1025_1027del, 1024_1028insKM)	this study	
pRK-2	pIGRK DNA was PCR amplified using oligo 3 and 4 (Table S2), BamHI digested
and ligated with BglII digested KM cassette (1255_1979del, 1254_1980insKM)	this study	
pRK-1_1	pRK-1 DNA was PCR amplified using oligo 5 and 6 (Table S2), and self-ligated 
(1982_2049del)	this study	
pRK-1ã	pRK-1 with oriã cassette inserted in SmaI site 	this study	
pRK-1_5ã	pRK-1 with AsuII /SpeI fragment replaced by oriã cassette digested with AsuII and SpeI	this study	
pRK-3	pIGRK DNA was PCR amplified using oligo 3 and 13 (Table S2), BamHI digested and ligated with BglII digested KM cassette (1979_1980insKM)	this study	
pRK-3_1	pRK-3 DNA was PCR amplified using oligo 5 and 6 (Table S2), and self-ligated 
(1982_2048del)	this study	
pRK-3_2ã	pRK-3_1 fragment (2048-7 bp pIGRK) )was PCR amplified using oligo 14 and 15 (Table S2), and cloned into pUC18HpaI SmaI site, subsequently obtained vector was HpaI digested and ligated with oriã cassette	this study	
pRK-3_3ã	pRK-3_2ã with AsuII site introduced between CR and IT1-4 elements using site directed mutagenesis and 19 and 20 oligo (Table S2) (2257A>C, 2258A>G)	this study	
pRK-3_4ã	pRK-3_3ã DNA was AsuII /HindIII digested, obtained  sticky ends were completely filled-in using Klenow fragment of E. coli DNA polymerase and then self-ligated (7_2258del)	this study	
pRK-3_5ã	pRK-3_3ã DNA was AsuII /EcoRI digested, obtained  sticky ends were completely filled-in using Klenow fragment of E. coli DNA polymerase and then self-ligated (2259_2348del)	this study	
Plasmid	Description		
pRK-1ã1	pRK-1ã  was GsuI digested, obtained  sticky ends were completely filled-in using Klenow fragment of E. coli DNA polymerase and then self-ligated (2290_2291del)	this study	
pRK-1ã2	pRK-1ã  with two MunI sites, introduced within CR element using site directed mutagenesis and 9 and 10 oligo (Table S2) (2236C>A, 2245T>C, 2247C>A, 2249G>T)	this study	
pRK-1ã3	pRK-1ã2 DNA was MunI digested, obtained  sticky ends were completely filled-in using Klenow fragment of E. coli DNA polymerase and then self-ligated	this study	
pRK-1_4	pRK-1 with 69 GTG repR codon replaced by GTC codon, obtained using site directed mutagenesis and 11 and 12 oligo (Table S2)	this study	
pUC-repR_1	pUC18 was EcoRI/BamHI digested and ligated with EcoRI/BamHI digested PCR product (1-883 bp pIGRK), amplified using 21 and 22 oligo (Table S2)	this study	
pUC-repR_2	pUC18 was EcoRI/BamHI digested and ligated with EcoRI/BamHI digested pRK-1_4 PCR product (1-883 bp pIGRK, 343G>C), amplified using 21 and 22 oligo (Table S2)	this study	
pUC-repR_3	pUC-repR_2 with BseRI/BbsI restriction fragment replaced by BseRI/BbsI restriction fragment from pBAD-repRÄH	this study	
pET-repR6H	pET28b+ with PCR amplified repR gene (using 44 and 45 oligo, Table S2) (141-880 bp pIGRK) cloned in NcoI and XhoI sites	this study	
pET-repR6HV69V	pET28b+ with PCR amplified repR gene (using 44 and 45 oligo, Table S2 and pUC-repR_2 DNA as a template, 141-880 bp pIGRK, 343G>C) cloned in NcoI and XhoI sites	this study	
pET-repR6HÄN	pET-repR6H digested with XbaI/HindIII, vector sticky ends were completely filled-in using Klenow fragment of E. coli DNA polymerase and then self-ligated	this study	
pETÄT7-repR6H	pET-repR6H digested with BglII /XbaI (deletion of T7 phage promoter), vector sticky ends were completely filled-in using Klenow fragment of E. coli DNA polymerase and then self-ligated	this study	
pET-repR6HM97L	pET-repR6H  with ATG codno (M97) replaced by CTT (L97) codon (434A>C, 436G>T), obtained using site directed mutagenesis and 41 and 42 oligo (Table S2)	this study	
pET-repR6HM100L	pET-repR6H  with ATG codno (M100) replaced by CTT (L100) codon (434A>C, 436G>T), obtained using site directed mutagenesis and 37 and 38 oligo (Table S2)	this study	
pET-PrepR6H	pET-repR6H  with EcoRV-HindIII fragment replaced by EcoRI-HindIII fragment from pRS-rk3	this study	
pBAD-repR	pBAD33ÄH with insertion of PCR amplified repR gene (using 46 and 47 oligo, Table S2 141-880 bp pIGRK) in XbaI and PstI sites (DNA sticky end was completely filled-in using Klenow fragment of E. coli DNA polymerase)	this study	
pBAD-repRÄN	pBAD-repR with XbaI/HindIII fragment deletion, sticky ends of digested vector were completely filled-in using Klenow fragment of E. coli DNA polymerase and then self-ligated	this study	
pBAD-repRV69V	pBAD-repR with BseRI/BbsI restriction fragment replaced by pUC-repR_2 BseRI/BbsI restriction fragment (69 GTG repR codon replaced by GTC codon)	this study	
pBAD-repRÄH	pBAD-repR was HindIII digested and obtained sticky ends were blunted by Mung bean nuclease (259_262del) and self-ligated	this study	
pRS-rk_1	pRS551 with pIGRK fragment (1-888 bp pIGRK PCR amplified using oligo 47 and 48, Table S2) cloned using EcoRI i BamHI	this study	
pRS-rk_2	pRS551 with pIGRK fragment (45-888 bp pIGRK PCR amplified using oligo 47 and 49, Table S2) cloned using EcoRI i BamHI	this study	
pRS-rk_3	pRS551 with pIGRK fragment (61-888 bp pIGRK PCR amplified using oligo 47 and 50, Table S2) cloned using EcoRI i BamHI	this study	
pRS-rk_6	pRS551 with pIGRK fragment (125-888 bp pIGRK PCR amplified using oligo 46 and 47, Table S2) cloned using EcoRI i BamHI	this study	
pRS-rk_7	pRS551 with pIGRK fragment (1-141 bp pIGRK PCR amplified using oligo 48 and 51, Table S2) cloned using EcoRI i BamHI	this study	
pRS-rk_9	pRS551 with PCR product (DNA fragment of pABB19PrepR amplified using oligo 15 and 16, Table S2 digested with EcoRI and BglII) cloned in EcoRI and BamHI sites	this study	
pRS-rk_10	pRS-rk_9 was digested with EcoRI (DNA sticky end was completely filled-in using Klenow fragment of E. coli DNA polymerase) and ligated withy BamHI digested PCR product (2260-888 bp pIGRK fragment amplified using oligo 47 and 52, Table S2)	this study	
pRS-rk_11	pRS-rk_9 was digested with EcoRI (DNA sticky end was completely filled-in using Klenow fragment of E. coli DNA polymerase) and ligated withy BamHI digested PCR product (2260-141 bp pIGRK fragment amplified using oligo nr 51 and 52, Table S2)	this study	
pRS-rk_12	pRS-rk_7 spontaneous mutant with IS1 insertion (76_77insIS1)	this study	
pRS-rk_8	pRS551 with pUC-repR_2 EcoRI/BamHI fragment cloned in EcoRI and BamHI sites	this study	
pRS-rk_28	pRS551 with pUC-repR_3 EcoRI/BamHI fragment cloned in EcoRI and BamHI sites	this study	
pRS-rk_29	pRS551 was digested with EcoRI and BamHI and ligated with PCR product (pUC-repR_3 fragment amplified with 47 and 50 oligo (Table S2)	this study	
pRS-rk_18	pRS551 with pUC-repR_8 EcoRI/BamHI fragment cloned in EcoRI and BamHI sites	this study	
Plasmid	Description		
pcI434R	pcI434 was digested with SalI and BamHI and ligated with SalI and BamHI digested PCR product (pRK-1_4 fragment, 137-880 bp pIGRK, 343G>C, amplified with 47 and 35 oligo, Table S2)	this study	
pcI22R	pcI22 was digested with SalI and BamHI and ligated with SalI and BamHI digested PCR product (pRK-1_4 fragment, 137-880 bp pIGRK, 343G>C, amplified with 47 and 35 oligo, Table S2)	this study	
pcI434R'	pcI434 was digested with SalI and BamHI and ligated with SalI and BamHI digested PCR product (pRK-1 fragment, 344-880 bp pIGRK, amplified with 47 and 36 oligo, Table S2)	this study	
pcI22R'	pcI22 was digested with SalI and BamHI and ligated with SalI and BamHI digested PCR product (pRK-1 fragment, 344-880 bp pIGRK, amplified with 47 and 36 oligo, Table S2)	this study	
pcI434RR'	pcI434 was digested with SalI and BamHI and ligated with SalI and BamHI digested PCR product (pRK-1 fragment, 137-880 bp pIGRK, amplified with 47 and 35 oligo, Table S2)	this study	
pcI22RR'	pcI434 was digested with SalI and BamHI and ligated with SalI and BamHI digested PCR product (pRK-1 fragment, 137-880 bp pIGRK, amplified with 47 and 35 oligo, Table S2)	this study	
pUC-RK4	pUC18 was digested with SmaI and ligated with PCR product (2049-2348 bp pIGRK fragment amplified using oligo 14 and 16 (Table S2)	this study	
pUC-RK3	pUC18HpaI  was digested with EcoRI and ligated with EcoRI digested PCR product (10-136 bp pIGRK fragment amplified using oligo 23 and 24 (Table S2)	this study	
pUC-RK_21	pUC18 digested with SmaI and ligated with annealed oligo 27 and 28 (Table S2) (5-42 bp pIGRK)	this study	
pUC-RK_22	pUC18 digested with SmaI and ligated with annealed oligo 25 and 26 (Table S2) (43-76 bp pIGRK)	this study	
pUC-RK4AsuII	pUC-RK4 with AsuII site introduced by site directed mutagenesis and 19 and 20 oligo (Table S2) (2257A>C, 2258A>G)	this study	
pUC-RK4_1	pUC-RK4AsuII digested with AsuII/EcoRI (2259_2348del), resulted sticky ends were completely filled-in using Klenow fragment of E. coli DNA polymerase and then self-ligated	this study	
pUC-RK4_2	pUC-RK4AsuII digested with HindIII/AsuII (2049_2257del), resulted sticky ends were completely filled-in using Klenow fragment of E. coli DNA polymerase and then self-ligated	this study	
pBAD33ÄH	pBAD33 was digested with HindIII, sticky ends were completely filled-in using Klenow fragment of E. coli DNA polymerase and then self-ligated	this study	
pUC18HpaI	pUC18 with HpaI site introduced by site directed mutagenesis and 17 and 18 oligo (Table S2) (419C>T, 420G>A, ÄAccI, ÄHincII, ÄSalI)	this study	
pABB19kan	pABB19 was digested with BamHI and ligated with BamHI digested PCR product (EZ-Tn5™ <KAN-2> transposone 20-1051 bp fragment, amplified using oligo 53 and 54, Table S2) 	this study	
pABB19PrepR	pABB19 digested with EcoRI and BamHI and ligated with EcoRI and BglII digested PCR product (1-141 bp pIGRK fragment, amplified using oligo 51 and 48 (Table S2)	this study
	
Cassette	Description		
KM cassette	pABB19kan fragment containing kanamycin resistance kan gene and transcriptional terminator (T) PCR amplified using oligo 15 and 16 (Table S2) containing BglII sites	this study	
oriã cassette	pDS132 fragment (1-392 bp) PCR amplified using oligo 7 and 8 (Table S2) containing SpeI and AsuII sites	this study	

The introduced mutations are described according to the following scheme: (i) deletions: 000_000del (nucleotide position of the first deleted pair of bases - nucleotide position of the last deleted pair of bases, deletion), (ii) insertions: 000_000insXYZ (nucleotide position of the first pair of bases above the insertion position of the first pair of bases below the insertion, ins - insertion; XYZ - name of the inserted element), (iii) nucleotide substitutions: 000X> Y (000X - position in the sequence and nucleotide occurring in the sequence originally, Y - the nucleotide introduced in its place), (iv) amino acid substitutions: X00Y (X - original amino acid, 00 - position in the sequence, Y - introduced amino acid). Sequence coordinates, unless otherwise stated, pIGRK (GenBank: AY543071.1), *unpublished data.
References
1	Di Lallo, G, Castagnoli L, Ghelardini P, Paolozzi L. A two-hybrid system based on  chimeric operator recognition for studying protein homo/heterodimerization in Escherichia coli. Microbiology. 2001;147:1651-1656.
2	Casadaban MJ, Cohen SN. Analysis of gene control signals by DNA fusion and cloning in Escherichia coli. J. Mol. Biol. 1980;138:179-207.
3	Platt R, Drescher C, Park SK, Phillips GJ. Genetic system for reversible integration of DNA constructs and lacZ gene fusions into the Escherichia coli chromosome. Plasmid. 2000;43(1):12-23.
4	Smorawiñska M, Szuplewska M, Zaleski P, Wawrzyniak P, Maj A, P³ucienniczak A, Bartosik D. Mobilizable narrow host range plasmids as natural suicide vectors ena-bling horizontal gene transfer among distantly related bacterial species. FEMS Microbiol Lett. 2012;326:76-82.
5	Bartosik AA, Markowska A, Szarlak J, Kuliñska A, Jagura-Burdz, G. Novel broad-host-range vehicles for cloning and shuffling of gene cassettes. J Microbiol Methods. 2012;88:53-62.
6	Dziewit L, Jazurek M, Drewniak L, Baj J, Bartosik D. The SXT conjugative element and linear prophage N15 encode toxin-antitoxin-stabilizing systems homologous to the tad-ata module of the Paracoccus aminophilus plasmid pAMI2. J Bacteriol. 2007;189(5):1983-97. 
7	Simons RW, Houman F, Kleckner N. Improved single and multicopy lac-based cloning vectors for protein and operon fusions. Gene. 1987;53: 85-96.
8	Zaleski P, Wawrzyniak P, Sobolewska A, Mikiewicz D, Wójtowicz-Krawiec A, Chojnacka-Puchta L, Zielinski M, P³ucienniczak G, P³ucienniczak A. New cloning and expression vector derived from Escherichia coli plasmid pIGWZ12; a potential vector for a two-plasmid expression system. Plasmid. 2012;67(3):264-71.
9	Guzman LM, Belin D, Carson MJ, Beckwith J. Tight regulation, modulation, and high-level expression by vectors containing the arabinose PBAD promoter. J Bac-teriol. 1995;177:4121-4130.
10	Philippe N, Alcaraz JP, Coursange E, Geiselmann J, Schneider D. Impro-vement of pCVD442, a suicide plasmid for gene allele exchange in bacteria. Plasmid. 2004;51:246-255.
