# Supplementary material for: Molecular dissection of the replication system of plasmid pIGRK encoding two in-frame Rep proteins with antagonistic functions
Source: BMC Microbiol. 2019 Nov 13;19:254. doi: 10.1186/s12866-019-1595-3 (PMC6854812; doi:10.1186/s12866-019-1595-3)
Supplement: Supplementary file 4 — Additional file 4: Table S3. Oligonucleotides used in this study (rtf). [file 12866_2019_1595_MOESM4_ESM.rtf]

Additional file 4

Table S3 Sequences of oligonucleotides used in this study. 
	Name	    Oligonucleotide sequence (5'→3')	
1	RKWTF 	GATGGATCCTTTCAACCCGTCTTTTTGGGTG	
2	RKWTR 	GATGGATCCCAACTTATACTACTGTTATATCCGG	
3	RK_B_F 	TAGGATCCACCGTGAGCAGTTCAGGA 	
4	RK_B_R	GAGGATCCCCCATATTGATTTTTCCTCATTGC	
5	RKssiF2	GTAGAATTCAGCAAAATCCTCCATAGCGAAG	
6	RKssiR 	TAGGAATTCGTTCACATTTTGAAGCGACGAAC	
7	r6kSpeIF 	GATACTAGTCCATGTCAGCCGTTAAGTGTTCC	
8	r6kAsuR 	GATTTCGAAGATCTGAAGATCAGCAGTTCAACCTG	
9	RKmutMMF	CTACTCTCTAACaATTGATATcAaTtGATTTTTAAAAAAGGCAGTTGTC	
10	RKmutMMR	GACAACTGCCTTTTTTAAAAATCaAtTgATATCAATtGTTAGAGAGTAG	
11	MVGTAF	ATCTATTTCCGAAGGTGTTGAATACGTcCATTTATTCCC	
12	MVGTAR	GGGAATAAATGgACGTATTCAACACCTTCGGAAATAGAT	
13	RK_Z_R	GAGGATCCTCACATTTTGAAGCGACGAAC	
14	DsoRK_R	CTAGAGTTGTCGGATTTGACAACCTC	
15	Pabbkasf	GTAAGATCTCGACGGCCAGTGAATTCGAGCTC	
16	Pabbkasr	GTAAGATCTGTTCCTGGCCTTTCCATGGATAATAGTTAACG	
17	pucHpaIR	GATCCTCTAGAGTtaACCTGCAGGCATGCAAGCTTG	
18	pucHpaIF	CAAGCTTGCATGCCTGCAGGTtaACTCTAGAGGATC	
19	RKASUIIF	CTCTAACCATTGATATTACTGGATTTTTcgAAAAGGCAGTTGTC 	
20	RKASUIIR	GACAACTGCCTTTTcgAAAAATCCAGTAATATCAATGGTTAGAG	
21	PRSPRKF	GCTGAATTCACTCTAGCCAGTTTCCAAGTAG	
22	pRKEPR	GCGGATCCTTATATTTCCGATTCAAAACCAAATTCG	
23	TNREPL	AAGAATTCAGACTCTAGCCAGTTTC	
24	TNREPR	CTGAATTCTCCATAGAAACCTCCTC	
25	PIGPRO2G	CAACTTTTTCTACTTTTTGCAACTTAATCTATTG	
26	PIGPRO2D	CAATAGATTAAGTTGCAAAAAGTAGAAAAAGTTG	
27	PIGPR01G	TAGCCAGTTTCCAAGTAGAAACTACAGTTTCTAAACTG	
28	PIGPR01D	CAGTTTAGAAACTGTAGTTTCTACTTGGAAACTGGCTA	
29	PMSUPEF	GTAGAATTCTTCTTATAACTAAGTTATAAAAAGTTG	
30	PmsrepMF	GTAGAATTCAAGTTGTAATCATGTATTGACTAG	
31	PmsBamHR	GATGGATCCATATTAAAACCCCATTAATAATTAAG	
32	Oligo d(G) 	GACCACGCGTATCGATGTCGACGGGGGGGGGGGGGGGG	
33	SP2RACE	CCTGTTAATTCGCAAAGTGCCTTTTGC	
34	SP3RACE	GCACGTATTCAACACCTTCGGAAATAG	
35	repRFSalI	GCGTCGACCATGGATATTGGAAATATATTAAATGAGAGTTTAAG	
36	repRpFSalI	GCGTCGACCATGCATTTATTCCCAAAAAATTTAAAAATTTTAATTAAAAAATATG	
37	PIGM2F	CGGAAATAATGGAGTCActtCTTTCACACG	
38	PIGM2R	CGTGTGAAAGaagTGACTCCATTATTTCCG	
39	SP1RACE	GATTCAAAACCAAATTCGAAATTTTCCTCTTCGTC	
40	PIGM1F	CGGAAATActtGAGTCAATGCTTTCACACG	
41	PIGM1R	CGTGTGAAAGCATTGACTCaagTATTTCCG	
42	M13pUCf	CCAGTCACGACGTTGTAAAACG	
43	M13pUCrFAM	FAM-AGCGGATAACAATTTCACACAGG	
44	pETRKCF	GACGCCATGGATATTGGAAATATATTAAATGAGAG	
45	pETRKCR	CGCTCGAGTATTTCCGATTCAAAACCAAATTCG	
46	BEcrepRF	GCTGAATTCGAGGAGGTTTCTATGGATATTGG	
47	PRSrepRR	GATGGATCCCGGTTTTATATTTCCGATTCAAAACC	
48	PRSPRKF 	GCTGAATTCACTCTAGCCAGTTTCCAAGTAG	
49	PRSPRKSS	GGTGAATTCACTTTTTCTACTTTTTGCAACTTAATCTATTG	
50	PRSPRKFs 	GCCGAATTCGCAACTTAATCTATTGACTAGTCC	
51	BamO3R	CTGGGATCCATAGAAACCTCCTCTTTTTATTTATTTC	
52	RKDR1F	AAAGGCAGTTGTCAAAAACTTCAACCGT	
53	KANBAM_F	GAGGGATCCCAACCATCATCGATGAATTG	
54	KANBAM_R	GAGGGATCCCTTCAACTCAGCAAAAGTTC	
55	RTkanF	GCCATTCTCACCGGATTCAGTCG	
56	RTkanR	GCGATTCCGACTCGTCCAACATC	
57	RTdxsF	GAAAGTCGCCGAATCGCTGAACG	
58	RTdxsR	CTTCTACGGTGACCAGCGCTTCA	

The underlined bolded fragments of the sequences indicate sites recognized by restriction enzymes attached to oligonucleotides. Positions of the changed nucleotides, introduced mutations are highlighted by small bolded fonts. FAM (fluorescein) attached to the 5'-end of the oligonucleotide.
