# Supplementary material for: Molecular dissection of the replication system of plasmid pIGRK encoding two in-frame Rep proteins with antagonistic functions
Source: BMC Microbiol. 2019 Nov 13;19:254. doi: 10.1186/s12866-019-1595-3 (PMC6854812; doi:10.1186/s12866-019-1595-3)
Supplement: Supplementary file 5 — Additional file 5: Figure S5. SDS-PAGE analysis of over-expression and purification of RepR(6His) and RepR’(6His) (rtf with Figure S5 in jpg format). [file 12866_2019_1595_MOESM5_ESM.zip › Additional file 5.rtf]

Additional file 5


Fig. S5 SDS-PAGE of recombinant Rep proteins purification procedure. A RepR(6His) purification, B RepR'(6His) purification. Lanes: (1) protein marker, (2) bacterial culture before induction, (3) bacterial culture after induction, (4) supernatant of centrifuged bacterial lysate, (5) proteins not attached to the Ni-NTA resin, (6, 7) washes, (8, 9) elutions.
